# Weak and unstable prediction of personality from the structural connectome

**DOI:** 10.1162/imag_a_00416

**Published:** 2025-01-03

**Authors:** Amelie Rauland, Kyesam Jung, Theodore D. Satterthwaite, Matthew Cieslak, Kathrin Reetz, Simon B. Eickhoff, Oleksandr V. Popovych

**Affiliations:** Department of Psychiatry, Psychotherapy and Psychosomatics, RWTH Aachen, Aachen, Germany; Institute of Neuroscience and Medicine, Brain and Behaviour (INM-7), Research Centre Jülich, Jülich, Germany; Institute for Systems Neuroscience, Medical Faculty, Heinrich-Heine University Düsseldorf, Düsseldorf, Germany; Lifespan Informatics and Neuroimaging Center, University of Pennsylvania Perelman School of Medicine, Philadelphia, PA, United States; Department of Psychiatry, University of Pennsylvania Perelman School of Medicine, Philadelphia, PA, United States; Penn-CHOP Lifespan Brain Institute, Philadelphia, PA, United States; Department of Neurology, RWTH Aachen University, Aachen, Germany; JARA-BRAIN Institute of Molecular Neuroscience and Neuroimaging, Forschungszentrum Jülich GmbH and RWTH Aachen University, Aachen, Germany

**Keywords:** structural connectome, diffusion-weighted MRI, big five personality traits, machine learning prediction analysis, cognition, individual differences

## Abstract

Personality neuroscience aims to discover links between personality traits and features of the brain. Previous neuroimaging studies have investigated the connection between the brain structure, microstructural properties of brain tissue, or the functional connectivity (FC) and these personality traits. Analyses relating personality to diffusion-weighted MRI measures were limited to investigating the voxel-wise or tract-wise association of microstructural properties with trait scores. The main goal of our study was to determine whether there is an individual predictive relationship between the structural connectome (SC) and the big five personality traits. To that end, we expanded past work in two ways: First, by focusing on the entire structural connectome (SC) instead of separate voxels and tracts; and second, by predicting personality trait scores instead of performing a statistical correlation analysis to assess an out-of-sample performance. Prediction of personality from the SC is, however, not yet as established as prediction of behavior from the FC, and sparse studies in this field so far delivered rather heterogeneous results. We, therefore, further dedicated our study to investigate whether and how different pipeline settings influence prediction performance. In a sample of 426 unrelated subjects with high-quality MRI acquisitions from the Human Connectome Project, we analyzed 19 different brain parcellations, 3 SC weightings, 3 groups of subjects, and 4 feature classes for the prediction of the 5 personality traits using a ridge regression. From the large number of evaluated pipelines, only very few lead to promising results of prediction accuracy*r*> 0.2, while the vast majority lead to a small prediction accuracy centered around zero. A markedly better prediction was observed for a cognition target confirming the chosen methods for SC calculation and prediction and indicating limitations of the personality trait scores and their relation to the SC. We therefore report that, for methods evaluated here, the SC cannot predict personality trait scores. Overall, we found that all considered pipeline conditions influence the predictive performance of both cognition and personality trait scores. The strongest differences were found for the trait openness and the SC weighting by number of streamlines which outperformed the other traits and weightings, respectively. As there is a substantial variation in prediction accuracy across pipelines even for the same subjects and the same target, these findings highlight the crucial importance of pipeline settings for predicting individual traits from the SC.

## 1 Introduction

Personality strongly affects interindividual differences of human behavior as well as personal strengths and vulnerabilities influencing all aspects of life (DeYoung et al., 2010). Personality neuroscience, therefore, aims to explore the neurobiological basis of personality. One of the most generally recognized and comprehensive models of personality is the Five-Factor Model (FFM), also known as the big five personality traits (R. McCrae & Costa, 1996). It describes five major dimensions of personality as openness to experience, conscientiousness, extraversion, agreeableness, and neuroticism. Scores for these dimensions are acquired from subjects by completion of self-report inventories.

From the neurobiological side, diffusion-weighted magnetic resonance imaging (dwMRI) measures the directional diffusion of water molecules in the brain (Mori & Barker, 1999) and enables both the*in-vivo*mapping of white matter (WM) tracts using tractography algorithms (Jeurissen et al., 2019) and the estimation of microstructural properties of the WM (Bihan et al., 2001). It therefore offers a diverse and novel insight into the human brain in addition to structural and functional MRI and opens up further options to investigate neural correlates of personality accordingly. Here, we aim to investigate whether there is an individual predictive relationship between the structural connectome (SC) of the brain calculated from dwMRI acquisitions and the big five personality trait scores.

A lot of prior studies have investigated neural correlates of personality extracted from structural or functional MRI (e.g.,Cai et al., 2020;Nostro et al., 2017). Regions and networks found to be related to personality have, however, differed between analyses. A large-scale quantitative meta-analysis conducted byY.-W. Chen and Canli (2022)on brain structural differences and personality did not find any replicable results of brain regions consistently related to personality traits and recommended looking into other modalities such as dwMRI for investigating the neurobiological basis of personality. While heterogeneous results are also common for functional MRI (fMRI) analysis investigating neural correlates of personality, a meta-analysis looking exclusively at the trait of neuroticism found consistent links between neuroticism and resting state fMRI activity in the left middle temporal gyrus, the left striatum, the right hippocampus, the left superior temporal gyrus, and the supramarginal gyrus (Lin et al., 2023).

Apart from the work on structural and fMRI, there has been previous work relating structural properties of the brain WM extracted from dwMRIs with the FFM personality traits (see below for references) which we would like to present here in more detail. The majority of these studies used a technique called tract-based spatial statistics (TBSS) (S. M. Smith et al., 2007). TBSS is a voxel-wise analysis of multi-subject diffusion data to relate different microstructural properties of the WM, which can be calculated using, for example, diffusion tensor imaging (DTI), to a certain target—here, personality trait scores. Most commonly, fractional anisotropy (FA) and mean diffusivity (MD) were estimated for the analysis. FA describes the fraction of the diffusion tensor that can be assigned to directionally dependent diffusion, and MD describes the average magnitude of diffusion in all directions (Bihan et al., 2001). However, results in these studies were quite heterogeneous.Leshem et al. (2019),Jung et al. (2010), andWilhelms et al. (2021)solely investigated the relationship with one trait (extraversion (Leshem), openness (Jung), and neuroticism (Wilhelms)) in different samples, and all reported statistically significant correlations for clusters of voxels and the respective personality trait. Other studies investigated all of the big five personality traits and reported statistically significant correlations only for a subset of traits. For example, microstructural properties of several WM voxel clusters were found to correlate only with openness, neuroticism, and agreeableness (Xu & Potenza, 2012), neuroticism (Bjørnebekk et al., 2013), conscientiousness (Booth et al., 2014), agreeableness, and conscientiousness (Rodriguez et al., 2019), or with all traits except openness (Sanjari Moghaddam et al., 2020). Furthermore, a recent study ofAvinun et al. (2020)reported null-findings for the relationship between mean FA across the brain and personality for all of the big five personality traits.

Alongside the voxel-wise analysis, other approaches applied special tractography algorithms to track and select major and known WM tracts for each subject (seeClayden et al., 2007for the specific algorithm used in the presented approaches) and calculate the average dwMRI microstructural properties along these tracts. The evaluated track properties can then be related to personality. WhileMcIntosh et al. (2013)investigated the relationship between the mean FA of 12 WM tracts and the traits extraversion and neuroticism,Lewis et al. (2016)studied how the FA of 10 WM tracts related to openness, agreeableness, and conscientiousness. Both studies found statistically significant correlations with at least one tract for each of the considered personality traits. However, in another study,Privado et al. (2017)only found a connection between openness and the FA of some of the extracted tracts. The reported results on the relationships between dwMRI data and personality traits thus remain very sparse and heterogeneous also for the tract-wise analyses.

The tract-based analysis of dwMRI data can be extended to the whole brain, where the entire SC can be calculated for each subject based on the whole-brain tractography (WBT) and a predefined parcellation of the gray matter (GM) (Sotiropoulos & Zalesky, 2019). In the first approximation, the SC is represented by an adjacency matrix, where each entry indicates whether any two regions of interest (ROIs) of the GM parcellation are connected with each other or not. The connectivity can then be defined in many different ways by weighting the SC adjacency matrix by using, for example, the number of streamlines (NOS) between two ROIs. Another SC weighting can also be the mean of any microstructural property, for example, MD or FA of all voxels that the streamlines connecting two ROIs pass through. So far, only a few studies have related graph metrics of local and global efficiencies and clustering coefficient of the SC to personality traits of extraversion, neuroticism, and openness to experience (Pang et al., 2019;Talaei & Ghaderi, 2022).

Overall, the available results on the relationships between dwMRI data and personality traits are rather fragmentary, heterogeneous, and controversial, which calls for additional and more systematic investigations of this problem for sufficiently large subject cohorts and high-quality neuroimaging data. All of the above studies have in common that they correlated dwMRI measures with personality trait scores and, therefore, do not offer any insight into the generalizability of their findings. On the other hand, predicting trait scores using machine learning (ML) algorithms in a cross-validation (CV) setting can, in contrast to statistical correlation analysis, give an indication of the out-of-sample performance and how generalizable certain findings and connections are (Dubois & Adolphs, 2016;Yarkoni & Westfall, 2017). Furthermore, the relationships between the SC itself and all five personality traits have so far not been thoroughly investigated and still await a systematic investigation. However, compared with the functional connectome (FC), the SC has been studied less as a predictor of individual phenotypes in general. In the process of calculating the SC from the diffusion MRI acquisition and predicting phenotypes from (the features of) the SC, there are many parameters that can each be adjusted in different ways. It has not yet been thoroughly investigated how the choice of brain parcellation, connectome weighting, feature selection of prediction, and CV algorithm may influence the prediction of one or another personality trait for one or another subject group, for example, males or females. While studies looking into these parameters did find differences between distinct settings, there are no clear best practices and parameter choices as there is a complex interplay between the different settings (Feng et al., 2022;He et al., 2022;Yeung et al., 2023).

The survey of related literature thus shows heterogeneous results for both the influence of different prediction pipeline parameters and the relationship between dwMRI measures and the big five personality trait scores. We, therefore, expected that prediction performance would vary considerably across pipelines. Furthermore, we expected that it would be more challenging to predict personality than cognition which shows a more consistent link to measures extracted from dwMRI (see e.g.,Dhamala et al., 2021;Feng et al., 2022;Yeung et al., 2023).

In this work, we, therefore, aimed to extend available results on the relationship between personality traits and dwMRI in two ways by making use of the entire SC instead of using a voxel-wise or tract-based analysis and by applying a predictive CV method instead of a statistical correlation analysis for an assessment of the out-of-sample performance. Considering that the SC has been studied less as a predictor of behavior than the FC, we further dedicated our study to evaluate a large number of different pipelines for the prediction of personality trait scores. We varied the brain atlas, the SC weighting, the subject group, and the feature class as well as feature selection process in order to unfold potential connections between the structural connectivity of the brain and personality and determine how they relate to different settings of the prediction process.

## 2 Materials and Methods

The goal of our study was to determine whether there is a general individual predictive relationship between features of the SC and the big five personality traits. Addressing such a question also requires investigating how different pipeline conditions may influence the prediction performance. We considered many different pipeline conditions given that there are no established best practices yet for setting these conditions when predicting from the SC. There have only been few studies investigating the results of different pipeline conditions and these studies show heterogeneous results (see e.g.,Feng et al., 2022;Yeung et al., 2023). Additionally, previous studies relating dwMRI measures to personality using voxel-wise or tract-based analysis showed heterogeneous results (seeSection 1), and evaluating many different pipeline conditions enabled us to provide a broader picture on whether and how personality and the SC are related and to ensure to not miss promising conditions. Overall, we compared the prediction results for 19 different cortical parcellations, 3 SC weightings, 3 subject groups, and 4 feature classes — 3 of them with additional conditions of feature selection — to predict 5 different personality traits. All these selections resulted in a total of 3,420 (19 x 3 x 4 x 5 x 3) different prediction pipelines that were repeatedly evaluated and compared for all considered cases of feature selection. An overview of the considered settings is schematically illustrated inFigure 1. Overall, we tried to investigate conditions that have shown to affect prediction accuracy in related studies and chose different options for these conditions based on options commonly applied in studies predicting phenotypes from the SC. The different conditions considered along the prediction pipeline and the rationale behind choosing these settings are described in more detail below.

**Fig. 1.**
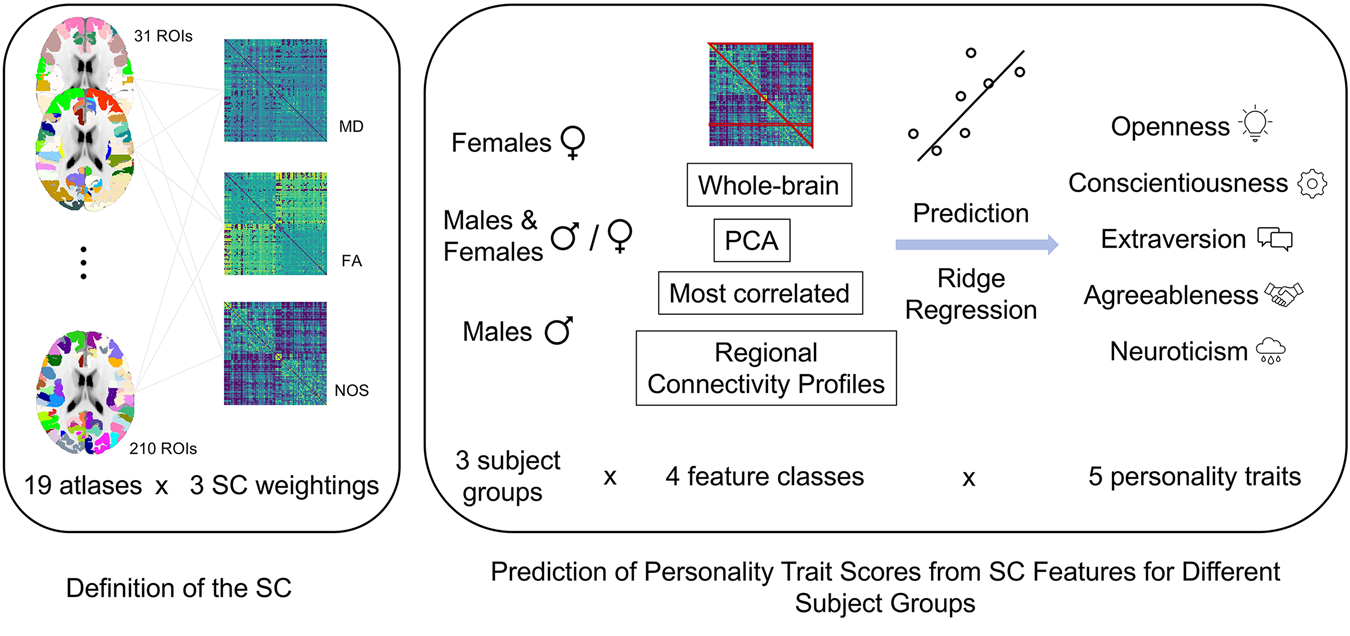
Overview of different options that were evaluated for predicting personality trait scores from the structural connectome (SC). For every subject, 19 parcellations were used to calculate SC from dwMRI for 3 different SC weightings given by the mean diffusivity (MD), fractional anisotropy (FA), and number of streamlines (NOS). The predictions of five personality traits were separately performed by ridge regression for three subject groups and four feature classes that included several conditions of feature selection such as number of features or a given region of used parcellation (see text for details).

We used the 19 different state-of-the-art cortical parcellations as described inDomhof et al. (2022). The parcellations were selected in a way to balance between parcellations derived from functional data and parcellations derived from structural data and provide a large variety of granularities from 31 to 210 ROIs. Detailed information on the different parcellations including names, number of ROIs, derivation, and references can be found inSupplementary Table S1.

For the connectome weighting, we considered the NOS connecting any two brain regions, calculated from the whole-brain tractography for a given parcellation (Jeurissen et al., 2019). We further employed the MD and the FA (Bihan et al., 2001) as connectome weighting. MD and FA are microstructural measures describing diffusion properties of the underlying tissue and offer additional information compared to the macrostructural NOS feature. MD describes the average magnitude of diffusion in all directions, and FA describes the degree of directionality of the diffusion.

Furthermore, we considered three subject groups: only females, only males, and a mixed-sex subject group. Such a setup of subject groups was chosen to evaluate whether there is an effect of sex on the personality prediction by SC. This consideration was motivated by previous findings, where an influence of sex on brain–personality correlations has been shown for GM volume (Nostro et al., 2017) and the resting-state FC (Nostro et al., 2018). Furthermore, the advantage of sex-specific machine learning pipelines has also been shown in other contexts (see e.g.,Alves et al., 2023;Gradus et al., 2021).

For prediction, features need to be selected from the SC. The most strait forward form feature class considers the entire SC (whole-brain). Depending on the granularity of the parcellation scheme, the number of features in this feature class in most cases exceeded the number of subjects in our dataset. This may lead to problems with overfitting and, therefore, motivated us to consider three additional feature classes that selected/calculated a much smaller number of features from the SC (details inSection 2.4).

### 2.1 MRI data and subject groups

The present study considered structural and diffusion MRI data from 560 healthy subjects from the Human Connectome Project (HCP) S1200 release dataset (Van Essen et al., 2013). The dataset includes diffusion imaging data for 972 individuals. However, there are many siblings and twins within the dataset, and we here decided to only use groups of unrelated subjects to not obscure results by possible family effects. The local ethics committee of the HCP WU-Minn Consortium gave its approval for the study and written informed consent was obtained from all subjects.

We created three subject groups of unrelated subjects. The first group consisted of n = 426 unrelated subjects (213 females, age 22–36 years, mean 28.5 ± 3.7). This corresponds to the highest number of unrelated subjects with diffusion data and personality trait scores available preserving an equal ratio of males and females. The second group consisted of n = 278 (age 22–36 years, mean 29.3 ± 3.7) unrelated males, and the third group was made up of n = 278 (age 22–37 years, mean 28.0 ± 3.8) unrelated females. There were overlaps between the mixed-sex group and the all-male and all-female groups, respectively. In total, considering the overlap between groups, data from 560 subjects was considered.

All data were acquired with a 3T Siemens PRISMA scanner. The T1-weighted (T1w) images were acquired at 0.7 mm isotropic resolution, the diffusion-weighted images (DWI) at 1.25 mm isotropic resolution with 90 gradient directions at each of 3 shells with b-values of 1,000, 2,000, and 3,000 s/mm^2^and a total of 18 b = 0 scans. The high-quality diffusion acquisition from a total of 270 directions on 3 different shells enables a more accurate estimation of the diffusion tensor and the microstructural properties derived from it, as well as the application of advanced methods for the estimation of fiber orientation distribution functions (fODFs) as a basis for tractography. For both T1w and DWI data, we used the minimally pre-processed images provided with the HCP dataset described in detail inGlasser et al. (2013).

### 2.2 SC calculation

For each subject, we calculated 57 SCs based on the 19 different parcellations and the 3 different connectome weightings based on NOS, MD, and FA (Fig. 1). All SCs were based on the same whole-brain tractography (WBT) for each respective subject. To calculate the WBT and extract the SC matrices, we used the pre-processed T1w and DWI data provided with the HCP dataset and an in-house pipeline optimized for parallel processing on high-performance computational clusters. The preprocessing included intensity normalization across runs,*topup*and*eddy*corrections, and gradient nonlinear correction. A detailed description of the HCP pre-processed data can be found inGlasser et al. (2013).

The pre-processed images were used for co-registration between the T1w and DWI spaces by FSL (S. M. Smith et al., 2004), as well as for the estimation of linear and non-linear transformation matrices from the standard MNI space to the native T1w space and vice versa. GM, WM, cortical/subcortical, cerebellar, and cerebrospinal fluid masks were generated in the DWI space as part of the registration process. Furthermore, MD and FA images were calculated from the DWIs for each subject using the*dwi2tensor*and*tensor2metric*functionalities of MRtrix3 (Tournier et al., 2019). After registration, the WBT was calculated. This part of the pipeline only used MRtrix3 functions. Shell- and matter-specific response functions were estimated using the*dwi2response dhollander*algorithm implemented in MRtrix3. Fiber orientation distribution functions (fODF) were then calculated using the multi-shell–multi-tissue constrained spherical deconvolution (MSMT-CSD) (Jeurissen et al., 2014) in each voxel of the DWIs. Subsequently, the WBT was calculated using an anatomically constrained probabilistic fiber tracking algorithm with second-order integration. Anatomically constrained tracking algorithms discarded streamlines ending in WM or CSF to obtain a more realistic set of streamlines (R. E. Smith et al., 2012). The WBT density was set to 10M streamlines, and other tracking parameters were set as follows based on the recommended default values in MRtrix3: step size = 0.625 mm, angle = 45 degrees, minimal length = 2.5 mm, maximal length = 250 mm, FOD amplitude for terminating tract = 0.06, maximum attempts per seed = 1,000, maximum number of sampling trials = 1,000, and downsampling = 3.

For all atlases, only cortical ROIs were selected to make the parcellations more comparable in this regard (given that the different atlases varied in the availability and extent of subcortical regions). However, as subcortical ROIs are known to be linked to emotion and other personality related measures such as memory (see e.g.,Fossati, 2012;LaBar & Cabeza, 2006), we repeated all analyses including subcortical ROIs. A detailed description of these additional experiments and the respective results can be found in the Supplementary Materials. The brain atlas images were sampled in the volumetric MNI152 nonlinear 6th generation standard space included in the FSL software package (S. M. Smith et al., 2004). To calculate the SC matrix from the WBT for a given parcellation, the atlas images were transformed to the native diffusion space for each subject using the pre-calculated transformation matrices. Then the labeled voxels within the GM mask were selected as seed and target regions to define streamlines connecting any two regions in the parcellation.

For the SC weighted by streamline count (NOS weighted), the number of streamlines connecting any two regions was entered in the corresponding cells of the connectivity matrix. No normalization by ROI size was applied to the NOS-weighted SC. For the SC weighted by MD and FA, additional steps were necessary. In the first step, the mean FA/MD per streamline was determined by taking the mean over all voxels that a respective streamline passes through. Then, to determine the final weights of the SC matrix, the mean FA and MD values were averaged across all streamlines connecting any two regions of a given parcellation. For all SC matrices, the diagonals containing self-connections were set to zero.

### 2.3 Personality trait scores

One of the most generally recognized and comprehensive models of personality is the Five-Factor Model (FFM) also known as the big five personality traits (R. McCrae & Costa, 1996). It describes five major dimensions of personality as openness to experience, conscientiousness, extraversion, agreeableness, and neuroticism. To obtain a score for each of the big five personality traits for each subject, all selected subjects filled out the English version of the 60-item version of the Neuroticism/Extraversion/Openness Five Factor Inventory (NEO-FFI) byR. R. McCrae and Costa (2004)as part of the Penn Computerized Cognitive Battery (Gur et al., 2010). There were 12 items for each of the 5 traits and all items were answered on a Likert scale ranging from 0 (strong disagreement) to 4 (strong agreement). The total score for each trait was obtained by adding up the responses from the separate items. Items keyed inversely were reversed before computation so that the total score for each trait was in the range [0, 48]. Additional information on the trait scores, such as their distributions and correlations of the traits between each other, can be found inSupplementary Figure S1.

### 2.4 Feature classes

Four feature classes were considered and derived from the SC for prediction of the personality trait scores (Fig. 1).

1. *Whole-Brain*: The first feature class was the entire upper triangle of the SC matrices (without diagonal). As the SC is symmetric, this included information from all structural connections considered in the respective parcellation.
2. *Most correlated (corr) edges*: The second class of features included a certain number*k*of SC edges that were most correlated with the personality traits across subjects. The selection of the most correlated edges was performed for the training set only and then applied to the test set to prevent data leakage.
3. *Principal component analysis (PCA):*The third feature class was composed of*k*first principal components (PC) as determined by a PCA of the SC matrices explaining most of the variance of the SC across subjects. To prevent data leakage, the PCA was fit on the training set only.
4. *Regional connectivity profiles (RCP)*: This feature class corresponded to separate rows of the SC reflecting the connectivity profile of a given brain region to the rest of the brain. For this case, every single row of the SC was evaluated as features.

Depending on the granularity of the parcellation scheme, the number of features in the whole-brain class in most cases exceeded the number of subjects in our dataset, which may lead to overfitting. This motivated us to consider the other feature classes above that contained a much smaller number of features, and that we compared with each other with respect to their prediction performance. For both the PCA and corr feature class, 19 different cases of feature selection were evaluated corresponding to different number of features*k*included in the analysis:*k*∈ {1, 2, 3, 4, 5, 10, 15, 20, 30, 40, 50, 60, 70, 80, 90, 100, 150, 200, #ROIs}. The latter number #ROIs corresponded to the number of brain regions of a given parcellation according to its granularity and was added to the feature selection cases of the corr and PCA classes in order to obtain the same number of features used in the RCP class. The RCP class also evaluated different cases of feature selection as each row of the SC was evaluated for the prediction. The number of different feature selection cases of the RCP feature class, therefore, depended on the number of ROIs of the considered parcellation.

### 2.5 Prediction

Taking into account the findings from the literature concerning the impact of used prediction algorithms on the prediction performance (Feng et al., 2022;Schulz et al., 2020;Yeung et al., 2023) (seeSection 4) and the computational burden of the different prediction models, we considered a simple ridge regression model for the prediction of personality trait scores from SC features. Ridge regression is a linear model that penalizes the regression coefficients based on their l2-norm and is defined as inEq. 1.

$$argmin_{\beta }\sum \;i{\left(y_{i}-\beta^{\prime }x_{i}+b\right)}^{2}+\alpha \sum_{k=1}^{K}\beta_{k}^{2}.\;$$ (1)

A nested 5-fold cross-validation (CV) was employed to tune the hyperparameter alpha of the ridge regression in the inner loop and assess the prediction performance on unseen subject samples in the outer loop. Alpha determines the strength of the l2-regularization, and the following values were evaluated in the inner loop of the nested CV: α in [0.001, 0.01, 1, 10, 50, 100, 500, 1,000, 5,000, 10,000]. The prediction performance was measured in terms of Pearson’s correlation between the predicted and empirical personality trait scores of the subjects in the test sets of the outer 5-fold CV loop. Then all five correlation values of the five different test sets were averaged such that a single prediction result was reported for a given CV procedure. The entire prediction procedure was repeated 100 times for different random splits of the data, which resulted in a distribution of prediction results (correlations) for each constellation of other conditions mentioned above. The 100 different random splits of the data were consistent across pipelines.

The SC features used for prediction were normalized before being used as input to the ridge regression using a global min–max normalization. The global maximum and minimum feature values from the training set were selected to scale all features to the range [0,1] as described inEq. 2:

$$x_{norm}=\frac{x-train_{min}}{train_{max}-train_{min}}$$ Eq. 2

where x is the feature used for training and prediction, and train_min_and train_max_are the minimum and maximum feature values calculated of the training set. For the PCA feature class, the normalization was applied to the SC before fitting the PCA on the data. For the other methods — whole-brain, most correlated edges, and RCPs — the features were first extracted from the SC, and only the selected features were then normalized and used to determine the minimum and maximum of the training set. As the distribution of the NOS was very skewed and stretched toward large NOS values, a log_10_function was applied to scale all edges with values >0 before the normalization was used to obtain a more symmetric and condensed NOS distribution without affecting small values too much.

### 2.6 Comparison of feature classes

To compare the different feature selection methods (feature classes), for any specific pipeline of*atlas, SC weighting, subject group, feature class, feature selection and target trait*, we selected the best result from all evaluated feature selection scenarios. For example, for the pipeline MIST31, NOS, mixed-sex, PCA and openness, we ran the pipeline for 19 different numbers of PCs. To compare this pipeline with the same pipeline with another feature class, we chose the number of PCs that led to the best prediction results, that was the largest mean prediction accuracy of the test set averaged over 100 random data splits used for CV procedure. The same was applied to the feature classes of the most correlated SC edges and the RCPs, where we chose the best prediction results with respect to the number of correlated edges and appropriate brain region, respectively. Using this method, for each feature class, we only had 1 distribution of test correlations from 100 random data splits that represented the obtained upper boundary of prediction accuracy for this feature class. This selection process did not have to be applied for the whole-brain feature class as there was only one option (whole SC) for the feature selection.

As the best number of PCs, best number of most correlated features and best RCP were chosen based on the prediction performance on the test set, these values represented an idealized performance estimate for each of the feature classes and might be overfit to this specific data set. For this reason, we performed additional calculations using another approach for the best feature selection. In this case, these variables were independently optimized in the inner CV loop of the nested CV, together with the regularization parameter alpha of the ridge regression. This resulted in only one distribution of prediction accuracy values calculated for the test set by the CV procedure for each specific pipeline with fixed atlas, SC weighting, subject sample, feature class, and prediction target. These prediction results can then be compared between the pipelines, for example, between feature classes. We only ran such a feature optimization on the mixed-sex subject group.

### 2.7 Prediction brain maps

For the RCP feature class, we can assign a prediction performance to every brain region of a given parcellation. Indeed, each case of the feature selection from the RCP feature class corresponded to selecting and using one row of the SC matrix for prediction, that was, the connectivity profile of one specific ROI (parcellated brain region) to the rest of the brain. Then the obtained prediction accuracy was assigned to all voxels of the selected region. For each parcellation, we iterated through all ROIs and created the whole-brain prediction map of that parcellation. Such a mapping procedure was repeated for all considered parcellations, and the obtained prediction maps were averaged across parcellations. This was done separately for all traits, SC weightings, and subject groups, which resulted in a total of 45 brain overlay maps reflecting the contribution of one or another group of voxels to the prediction of personality traits.

### 2.8 Prediction of cognition

To obtain context to the results for the personality prediction, we additionally predicted a cognition variable provided with the HCP S1200 dataset. We chose the age-adjusted total composite score from the NIH Toolbox Cognitive Function Battery (Gur et al., 2010), which included scores from picture vocabulary, reading, flanker, dimensional change card sort, picture sequence memory, list sorting, and pattern comparison tests, and comprises both fluid and crystallized cognition measures. For these experiments, we only used the larger mixed-sex subject group. There was no significant correlation of the cognition score with age or sex across the considered subjects (r_Cognition-Sex_= -0.014, r_Cognition-Age_= -0.055).

## 3 Results

We applied a ridge regression model to predict personality trait scores from features of the SC evaluating many different pipelines to (1) investigate the predictive relationship between personality and the structural connections of the brain and (2) evaluate the influence that different settings in the prediction pipeline have on the prediction accuracy.

Below we first present a few examples of prediction results for varying the feature class and feature selection before giving an overview of the results from all different pipelines. Then we discuss the prediction brain maps illustrating how the connections from groups of voxels participate in prediction and, finally, evaluate and illustrate the influence of different pipeline parameters on prediction accuracy.

### 3.1 Prediction performance

#### 3.1.1 Personality trait prediction

**Fig. 2.**
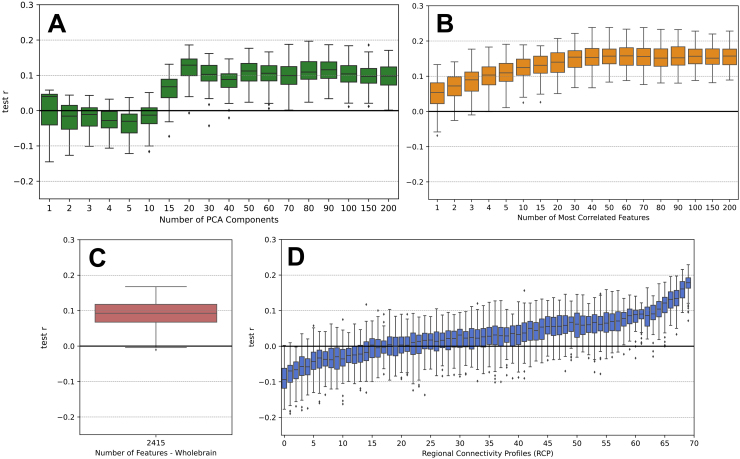
Examples of prediction results for all four feature classes. The calculations were performed by the pipeline with the Desikan–Killiany atlas (70 ROIs), NOS weighting, mixed-sex subject group, and the trait openness. The box plots show the distributions of the prediction accuracy as given by Pearson’s correlation between the predicted and empirical personality scores obtained for the test sets over 100 random splits of the data of the 5-fold cross-validation. The prediction results are depicted for (A) the PCA feature class with different number of PCA components, (B) the corr feature class with different number of the most correlated features, (C) the whole-brain feature class, and (D) the Regional Connectivity Profile (RCP) feature class for different brain regions, that is, rows of the SC matrix. RCPs are sorted by the mean prediction accuracy. The respective conditions of the feature selection are indicated on the horizontal axes with non-linear scaling in plots (A) and (B).

The prediction performance of personality traits from the DWI data is illustrated inFigure 2for a few examples of the feature class and feature selection. The shown results were obtained using the Desikan–Killiany atlas (Desikan et al., 2006) with 70 ROIs, NOS weighting of the SC, the mixed-sex subject group, and the trait openness as target variable (see Methods). Overall, we can see that the prediction accuracy as given by the correlation between predicted and empirical openness scores does not on average reach values higher than ~0.19 for this set up and for all feature classes and feature selections considered. In particular, for the feature class*PCA*and varying the different number of PCs (Fig. 2A), a larger number of PCs (~20) must be used to obtain a positive prediction accuracy, which then stays approximately constant at ~0.1 when adding further PCs. For another feature class referred to as “*corr*” of the most correlated features (see Methods), the prediction accuracy reached plateaus of ~0.15 at around 40 of the most correlated SC edges (Fig. 2B). However, when considering the*whole-brain*feature class (Fig. 2C), which used all SC edges leading to 2,415 features in total including the edges of lower correlation, the accuracy was reduced to ~0.1, which indicates that the plateau seen for the corr feature class (Fig. 2B) is expected to decrease again when more SC edges are added as features into the prediction process. Finally, the best accuracy of the considered setup was observed for the RCP feature class, when the connectivity profiles of individual brain regions to the rest of the brain were selected as features (Fig. 2D). For the RCP feature selection, the prediction accuracy depends substantially on the selected RCP, which can be observed by the strongly varied correlation values across brain regions. Nevertheless, the RCP features that may include SC edges with high and low correlations with the target score as compared with the corr feature class had the potential to outperform the latter and other feature classes, when an appropriate RCP was chosen. An in-depth comparison of the different feature classes follows inSection 3.3.4.

**Fig. 3.**
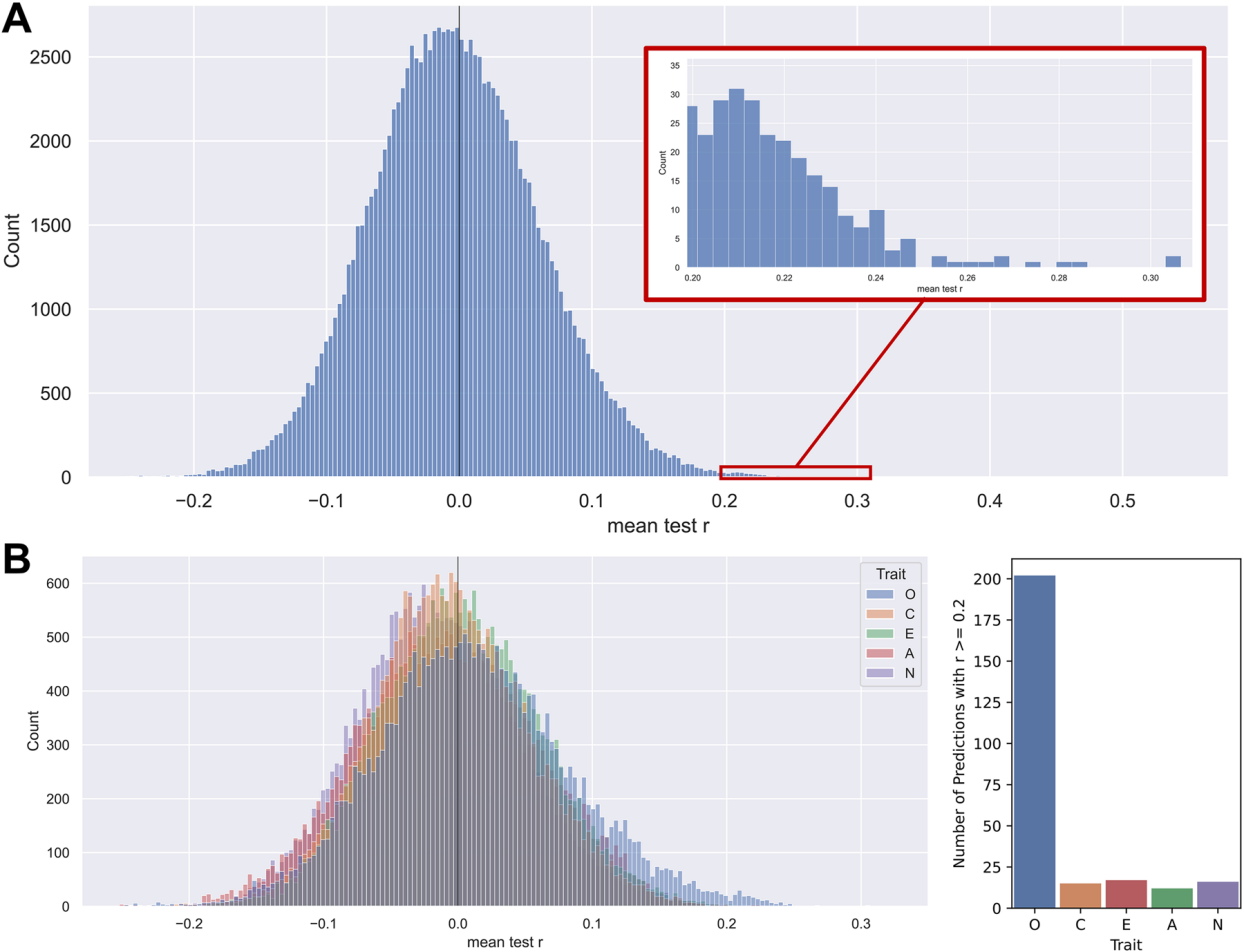
Overview of all results on personality prediction by structural connectome. (A) Histogram showing the overall distribution of the prediction accuracy given by the mean correlation*r*between empirical and predicted personality traits obtained for the test sets and averaged across 100 random subject splits for cross-validation. The prediction results are collected from all different pipelines with varying parcellation, SC weighting, subject group, personality trait, and feature class as well as all conditions of the feature selection. The latter includes different number of PCA components (19 options), different number of the most correlated SC edges (19 options), and all RCPs (number of options depended on the granularity of the parcellation). The histogram in total contains 123,526 average prediction accuracies. The selected and enlarged area of the histogram shows the cases of*r*> 0.2. (B) Left: Five overlaid histograms of the prediction results from plot (A) separated by the five different personality traits as indicated in the legend. Right: Number of occurrences of the different personality traits in the pipelines leading to prediction accuracies*r*> 0.2, marked by the red box in A. The traits are indicated on the horizontal axis. The trait abbreviations are as follows O: Openness, C: Conscientiousness, E: Extraversion, A: Agreeableness, N: Neuroticism.

A second example is illustrated inSupplementary Figure S2. The presented results were obtained using the Shen atlas (Shen et al., 2013) with 79 ROIs, the NOS weighting of the SC, the mixed-sex subject group, and the trait neuroticism. Compared with the first example, the parcellation and the trait were exchanged for another condition. Here, we see overall lower prediction accuracies. For the whole-brain, corr, and PCA feature class, only negative correlations were obtained (Supplementary Fig. S2A–C). Solely for the RCP feature class, some few RCPs led to positive correlations (Supplementary Fig. S2D). The proportion of RCPs leading to positive values as well as the maximum achieved correlation lies below that found for the RCP feature class in the first example (Fig. 3D).

#### 3.1.2 Distribution of all prediction results

In this study, we performed a large number of prediction calculations for 3,420 different pipelines reflecting a combination of 19 atlases x 3 SC weightings x 3 subject groups x 4 feature classes x 5 target traits (see Methods). Every pipeline was executed several times using different conditions for the feature selection of a given feature class such as different number of PCs (PCA class) and the most correlated edges (corr class), and different brain regions (RCP class), seeFigure 2. By this, the total number of prediction results accumulated to 123,615, which were obtained by averaging over 100 random splits of the subjects into 5-fold CV. These average results therefore required a 100-fold amount of individual prediction runs consisting of a nested 5-fold CV loop used to tune the hyperparameter of the ridge regression and calculate the prediction accuracy on the test sets.

To get an overview over the prediction performance of all evaluated pipelines, we illustrate the distribution of the average prediction accuracies from all conducted calculations inFigure 3A. With only a few exceptions, the prediction results over all different settings yielded low correlations for the prediction of personality trait scores from the SC features. The mean of the overall distribution of the average prediction accuracies was around zero at*r*≈ -0.003. Some very few prediction pipelines led to promising prediction accuracies of*r*> 0.2 (Fig. 3A, enlargement), which are in the range of values reported in the literature for prediction of personality traits from FC (Cai et al., 2020;Dubois et al., 2018;Nostro et al., 2018). Information on which exact combination of pipeline settings led to which prediction accuracy is shared as csv files uploaded to the project’s GitHub repository (https://github.com/ameliecr/SCandBigFive).

The grand collection of all prediction results inFigure 3Acan be split into subgroups to reveal whether one or another case of the pipeline conditions may lead to different prediction performance than the others. For example, we may compare the prediction results for different personality traits and split the overall result distribution into five subgroups of specific traits, while the other pipeline conditions vary within every subgroup. The distribution histograms reflecting the prediction accuracy for the five personality traits are illustrated in the left plot inFigure 3B. Here one can observe that, despite the overall low prediction performance, the highest prediction accuracies were achieved for the trait openness. The mean correlations of the different distributions all still remain close to zero with the highest mean correlation for the openness trait at*r*= 0.012, shifted slightly to the right. The effect size in terms of Cohen’s d was small to medium when comparing the result distribution of openness with the other traits: d_O-A_= 0.28 (Cohen’s d between the distributions for the openness (O) and agreeableness (A) trait), d_O-C_= 0.26, d_O-E_= 0.15, d_O-N_= 0.40. No notable differences in predictability could be observed among the other traits, as it is difficult to compare the overall very low effect sizes found for the other traits except for d_E-N_= 0.28. Furthermore, we observed a much larger proportion of the best prediction results with*r*> 0.2 for the openness than for the other traits (Fig. 3B, right). We also found that especially good results can be reported for this trait in the female-only subject group, but also in the mixed-sex subject group which is shown inFigure 7.

#### 3.1.2 Cognition prediction

**Fig. 4.**
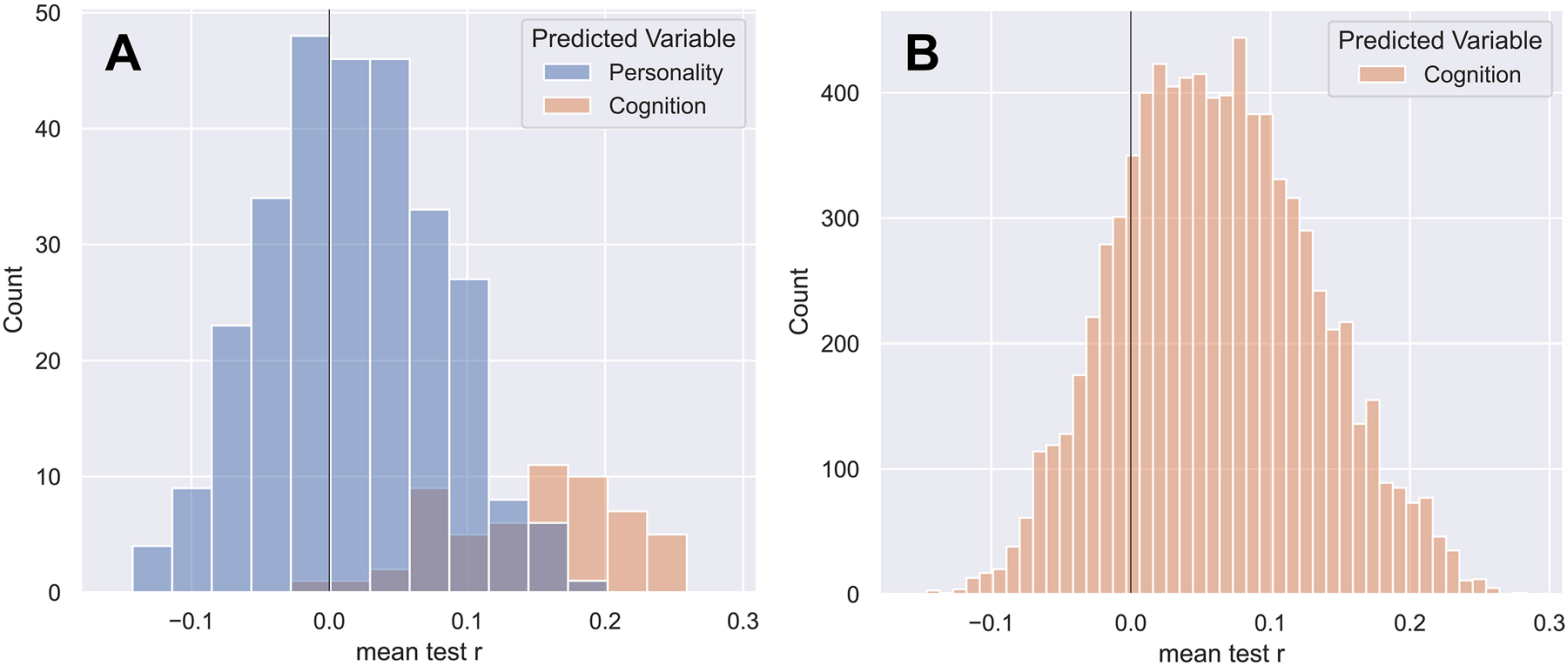
Results for the prediction of cognition. (A) Comparing the prediction results for personality traits and cognition. The distributions of the prediction accuracy (correlation) for cognition and all five personality traits are shown as indicated in the legend. In both cases, the same prediction pipeline and DWI data were used of the mixed-sex group (n = 426), the entire SC (whole-brain feature class), and varied parcellations and connectome weightings. (B) The prediction accuracy of all evaluated prediction pipelines for cognition. Compared with (A), this distribution includes results for all four feature classes (instead of only the whole-brain feature class) and all of the evaluated feature selection options.

To provide an additional context for the prediction results of personality traits illustrated inFigure 3, we also considered a cognition score from the HCP dataset as another prediction target. We, therefore, calculated the prediction accuracy for this score for the mixed-sex subject group, and varied brain parcellation, SC weighting, and feature class. The obtained correlation distribution for the whole-brain feature class is depicted inFigure 4Atogether with that of all five personality traits for the same conditions of the prediction pipeline. We chose the whole-brain feature class since this does not have the additional variable of the feature selection, that is, different RCPs or a different number of PCA and corr features, which facilitates the comparison. For completeness,Figure 4Bshows prediction accuracies for all feature classes and their evaluated feature selection options for the prediction of cognition. One can see that predicting cognition led to slightly higher values of the prediction accuracies than predicting personality trait scores did (Fig. 4A). Interestingly, the mean of the accuracy distribution for cognition prediction lies at about*r*= 0.15, whereas the prediction accuracy for personality traits is distributed around*r*= 0.02 for the pipelines considered inFigure 4A. The difference between the two distributions shows a very high effect size (Cohen’s d) of 2.06. Furthermore, the highest obtained correlation for cognition was*r*= 0.28, while it was*r*= 0.21 for openness,*r*= 0.23 for conscientiousness,*r*= 0.19 for extraversion,*r*= 0.20 for agreeableness, and*r*= 0.21 for neuroticism. This demonstrates that the used prediction pipeline and DWI data can in fact lead to reasonable prediction results for cognition, where the accuracy distribution was clearly shifted to positive values (Fig. 4A). This was, however, not the case for predicting personality traits (Figs. 3and4A), where the overall and the best-case correlations were smaller than those for cognition.

### 3.2 Prediction brain maps

In addition to evaluating the prediction accuracy, we are interested in exploring which brain regions actively participated in personality prediction. We address this question for the prediction results obtained from the pipelines using the RCP feature class. For this, we calculated the prediction brain maps of the mean prediction accuracy averaged over all considered parcellations as described inSection 2.7in Methods.

**Fig. 5.**
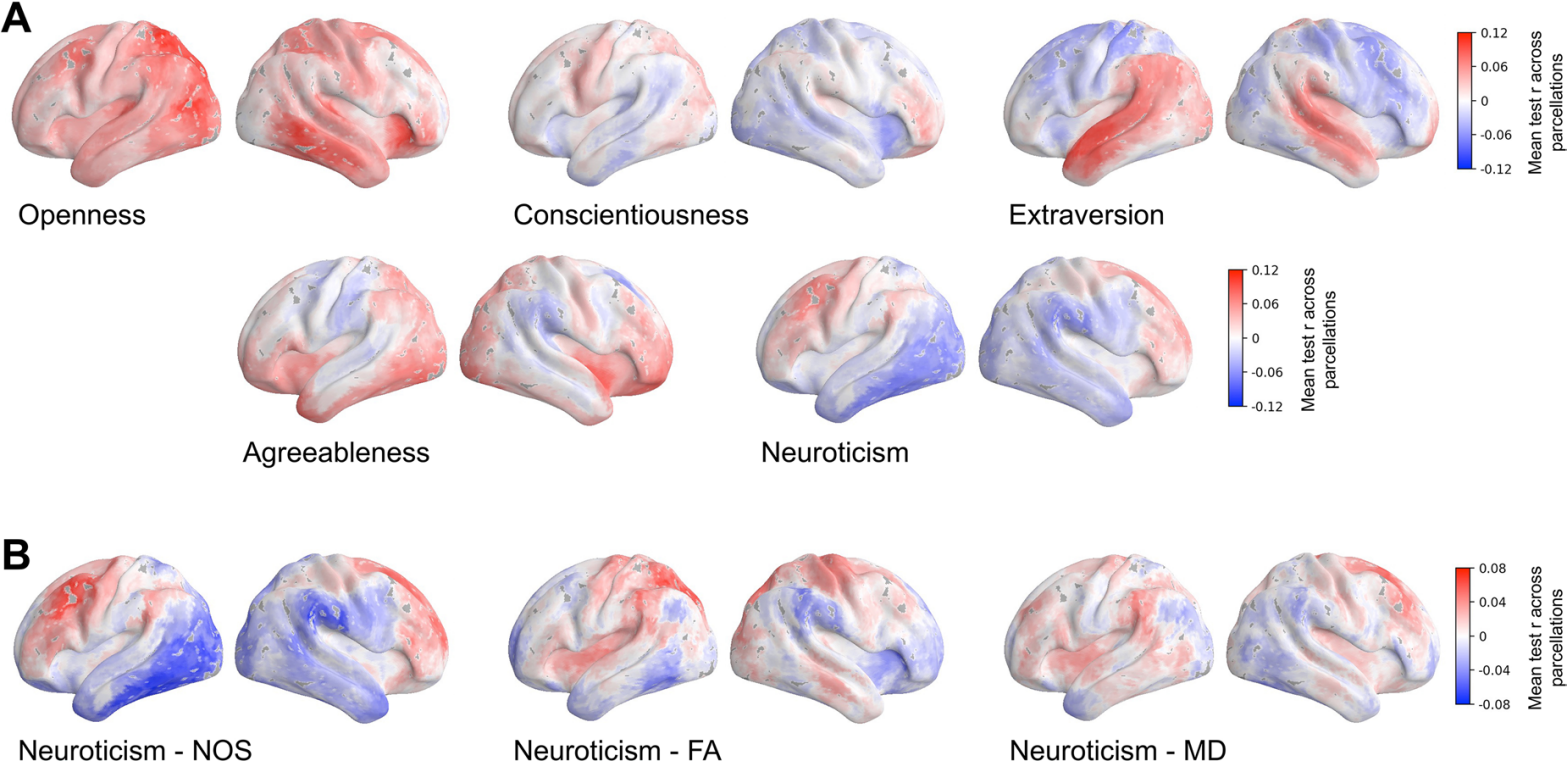
Prediction brain maps associated with the performance of personality prediction from DWI data. The prediction accuracy (correlation) of the RCP feature class was assigned to all voxels of the respective brain regions and averaged over all considered parcellations for fixed other conditions of the prediction pipeline (see Methods). The brain maps are illustrated for (A) the five different personality traits indicated in the plots as prediction targets obtained for the mixed-sex subject group and the NOS SC weighting, and (B) the three different SC weightings indicated in the plots obtained for the mixed-sex subject group and the personality trait neuroticism. The average correlation is depicted by colors with the scaling indicated in the color bars. Left and right hemispheres are shown from the lateral view. Abbreviations are NOS: the number of streamlines, MD: the mean diffusivity, and FA: the fractional anisotropy. The visualization of the maps was created using the neuromaps toolbox (Markello et al., 2022) including the volume-to-surface transformations as proposed and defined in (Buckner et al., 2011;Wu et al., 2018).

Figure 5shows exemplary maps for different traits and different SC weightings. Voxels in red show that, across all parcellations, this voxel belonged to an ROI for which the respective RCP led to an average positive test set correlation. The “stronger” this red color is, the higher the positive test set correlation. Voxels in blue indicate that, on average across all parcellations, these voxels belonged to ROIs for which connections to all other parts of the brain led to a negative test set correlation. That is, no generalizable connection between the RCP and the personality trait score was found. The stronger the blue, the more negative the test set correlation was. We do want to clarify that one cannot say that a stronger negative average correlation corresponds to a “worse” prediction than a slightly negative average correlation. Correlations*r*< 0 indicate an unsuccessful prediction but it is unclear how negative prediction accuracies relate to each other.

We found that predicting the five different target traits was associated with different prediction brain maps as illustrated inFigure 5Afor the NOS weighting and mixed-sex subject group. In particular, the map for openness stands out as it indicates that, especially for the left hemisphere, RCPs from almost all regions of the brain actively participated in the prediction of this trait and delivered positive prediction accuracy across different parcellations. This may be compared with the conscientiousness map exhibiting a uniformly low average correlation (low color intensity inFig. 5A, middle), which makes it difficult to select any brain areas clearly associated with this trait. On the other hand, the well-pronounced landscapes of high and low average prediction accuracies in the maps for extraversion, agreeableness, and neuroticism can help to distinguish brain regions whose connections are important for predicting the respective trait (Fig. 5A).

The discussed prediction brain maps can also be calculated for the three SC weightings as illustrated inFigure 5Bfor the mixed-sex subject group and the trait neuroticism. Here we also observe that different SC weightings can be associated with different brain maps. In particular, the NOS weights were accompanied by a distinct landscape of the brain map as compared with those of MA and FA weightings (Fig. 5B). For the NOS weighting, one can clearly distinguish the brain areas positively contributing to the personality prediction from those with a little or even negative impact on the prediction performance (Fig. 5B, red and blue domains). The other two SC weightings based on FA and MD led to less contrasted prediction brain maps that are rather similar to each other, but distinct from the NOS case, which we consider in more detail later below.

It must be noted that the maximal mean correlations observed in the maps are low at*r*= 0.12 when comparing traits and*r*= 0.08 when comparing weightings. Nevertheless, the obtained brain maps can be compared with baseline maps of “random prediction” (Supplementary Fig. S3), where we additionally ran permutation tests for the RCP feature class. There, we again performed 100 repetitions of a nested 5-fold CV for the same pipelines, but the target values were randomly shuffled between subjects. The baseline maps for the different traits show a mean correlation across parcellations close to zero for the entire brain (Supplementary Fig. S3). Therefore, it can be reported that distinct brain maps arise for the different prediction targets and connectome weightings marking RCPs which lead to positive or negative prediction accuracies across all 19 parcellations. This shows that it depends on the prediction target and the considered weighting for which RCPs can predict the personality target more or less accurately, that is, which brain areas and their connections might be more or less related with the trait of interest.

### 3.3 Influence of different pipeline settings

#### 3.3.1 Parcellation

We observed no clear trend toward a superiority of certain brain parcellations for personality prediction. There seems to be a slight tendency toward an advantage of a finer granularity (more brain regions) within the same parcellation scheme, for example, when the best prediction results were selected from the RCP feature class (Supplementary Fig. S5). A similar trend can be observed when considering pipelines leading to correlations*r*> 0.2 for the prediction of cognition. Here, parcellations with higher granularities more often lead to such results than coarser parcellations (Supplementary Fig. S6B). When considering all different pipelines, it however depends on the combination of the individual settings of the pipeline conditions which brain parcellation performs best.

Adding subcortical ROIs to the parcellations had neither positive nor negative notable effects on prediction results (Supplementary Fig. S4). When comparing the mean values of the accuracy distributions for cortical only and cortical + subcortical parcellations, there is only a very small shift with a small effect size in terms of Cohen’s d between the two distributions (mean_cort_= -0.003, mean_cort+subcort_= -0.004, d = 0.0158). While the very best result improved from r_max,cort_= 0.307 to r_max,cort+subcort_= 0.329, when including subcortical ROIs, the number of results with*r*> 0.2 decreased from n_r>0.2,cort_= 262 to n_r>0.2,cort+subcort_= 247. A detailed analysis of the results can be found in theSupplementary Materials.

#### 3.3.2 Connectome weighting

**Fig. 6.**
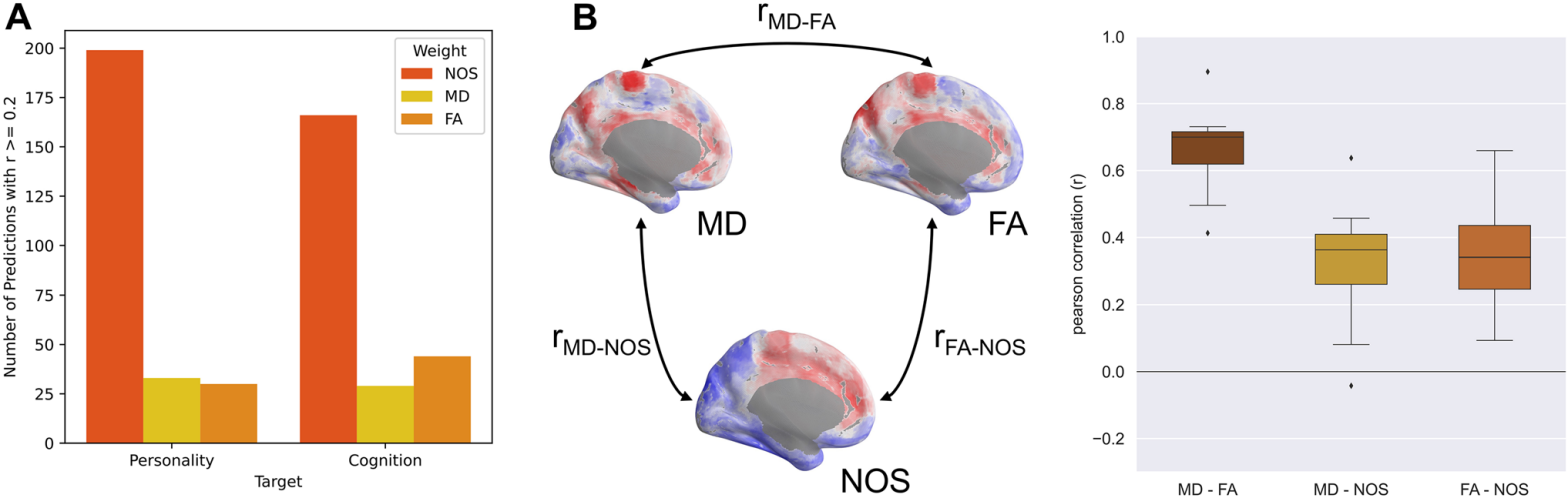
Impact of connectome weighting on prediction of personality and cognition. (A) Fraction of the predictions results with the prediction accuracy*r*> 0.2 broken down by the different connectome weightings as indicated in the legend for both the prediction of personality and cognition as denoted on the horizontal axis. (B) Comparison of the prediction brain maps obtained for different connectome weightings and the RCP feature class, seeFigure 5B. The maps for different weightings were compared by correlation across voxels for all 5 personality traits and all 3 subject groups, so that the boxes in the graph on the right each represents a distribution of 15 different correlation values. Abbreviations are NOS: the number of streamlines, MD: the mean diffusivity, and FA: the fractional anisotropy. The example maps on the left show maps for the trait neuroticism and the mixed-sex subject group.

We also did not clearly observe an overall best connectome weighting for the prediction of personality traits. The prediction performance of the weighting depends on the other settings, such as the parcellation scheme, the trait, or the feature class. For predicting cognition, we can, however, see a quite clear difference between the different SC weightings. NOS performs better than FA which in turn performs better than MD (Supplementary Fig. S7). Furthermore, among the best prediction results, characterized by an average prediction accuracy of*r*> 0.2, NOS is the weighting occurring most frequently for both cognition and personality traits. In particular, ~69.5% and 76% of the best pipelines leading to prediction accuracies of*r*> 0.2 used NOS as SC weighting for the prediction of cognition and personality traits, respectively (Fig. 6A).

The properties of SC weightings and their impact on the prediction results can also be compared based on the prediction brain maps calculated for the RCP feature class by averaging across all considered parcellations (Fig. 5B). We calculated the correlations between any of these two maps, which was repeated for all combinations of different personality traits and subject groups to obtain distributions of the correlations (Fig. 6B, right). One can see a high similarity (correlation) between the cortical maps from the MD and FA weighting, whereas the brain maps of both microstructural DWI weightings exhibit distinct patterns as compared with that of the NOS weighting, which is reflected by a low correlation of NOS maps with both MD and FA maps. Considering that the meaning of more or less negative correlations is unclear but is given a meaning by correlating the maps of different weightings, we further correlated the maps after setting all voxels with*r*< 0 to zero and only considering voxels with*r*> 0 for all three weightings. While these configurations might in turn have other drawbacks, the stronger similarity between maps of MD and FA weighting and the more distinct pattern of the NOS map were confirmed for all three setups. This also holds for individual parcellations for both personality traits and cognition (Supplementary Fig. S8B and C). Analogous similarity patterns can also be observed when calculating the correlation between the differently weighted normalized SCs for all subjects selected for the study (Supplementary Fig. S8A). Correlations between the FA- and MD-weighted SCs are close to 1, whereas correlations between NOS-weighted connectomes and the others are significantly lower for all parcellations except for the parcellation with the coarsest granularity (MIST 31) (Supplementary Fig. S8A).

#### 3.3.3 Subject group

**Fig. 7.**
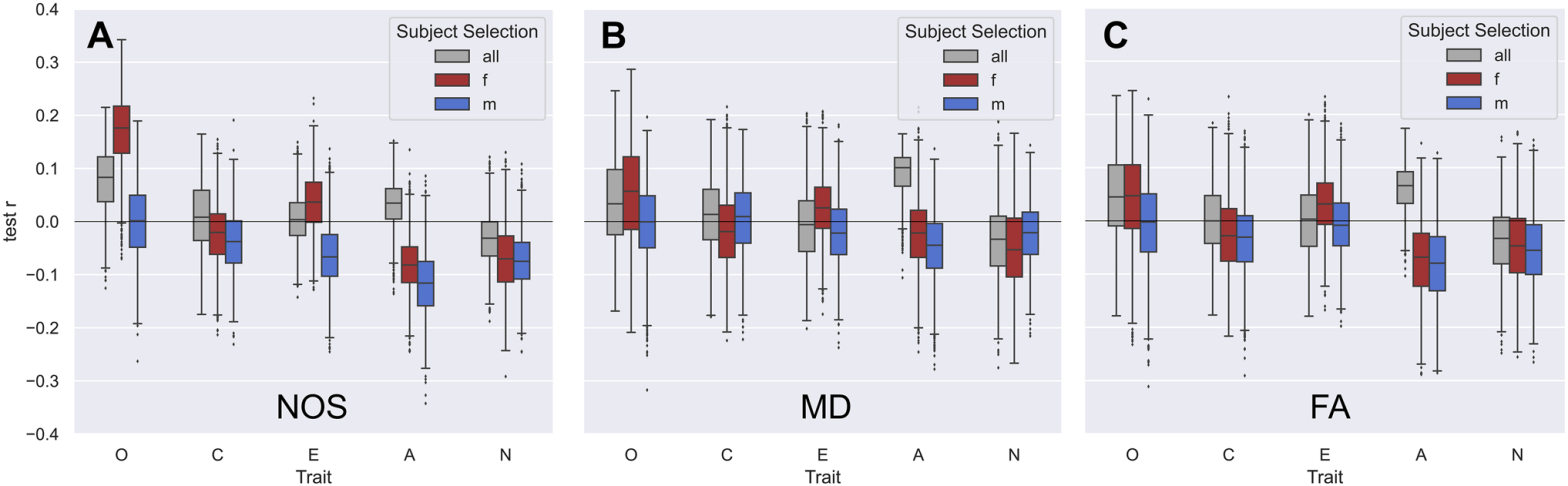
Impact of different subject groups on the prediction results. The plots include results from the whole-brain feature class and all different parcellations for (A) the NOS weighting, (B) the MD weighting, and (C) the FA weighting. In each plot, the results are separated by trait as indicated on the x-axis and the different subject groups are indicated in the legend—all: mixed sex, f: females, m: males.

In this study, three different subject groups of males, females, and mixed-sex subjects were considered as one of the pipeline conditions. We observed some differences in terms of prediction accuracies between these subject groups, which were, however, not consistent across different pipeline settings such as different SC weightings and different traits.Figure 7shows prediction accuracies from the pipelines using the whole-brain feature class for the different subject groups across the five different traits for the NOS weighting (Fig. 7A), the MD weighting (Fig. 7B), and the FA weighting (Fig. 7C). For the NOS weighting, the female subject group performs a lot better than the mixed-sex and male groups for the openness trait. This is, however, not the case for openness using the MD or FA weighting. For most other traits, there is no improvement when separating the subjects by sex or even a deterioration such as for the trait agreeableness for all three SC weightings. As the prediction accuracies and their differences were overall low, it is little conclusive whether the differences between subject groups arise from distinct brain–personality relationships in males and females or simply from the fact that these groups were composed of different subjects independently of subject sex. Further investigations would be necessary to ensure there is a difference if a more stable SC–personality connection could be established.

#### 3.3.4 Feature classes

An important pipeline condition that can influence the prediction performance is the employed feature class. The prediction results obtained for different feature classes were compared in this study in two different ways, seeSection 2.6in Methods. According to the first approach, we calculated the prediction accuracies on the test set for all considered cases of pipeline conditions and feature selections. Then the best prediction results were selected for each of the feature classes across the feature selection instances, that is, the best number of most correlated features, the best RCP, and the best number of PCs. InFigure 2A, B, and D, for example, only the distribution with the highest mean value is chosen for each feature class, respectively, such that they can be compared with each other and the distribution ofFigure 2Cfor the whole-brain feature class. This procedure was repeated for each combination of other pipeline settings. In this comparison, the RCP feature class outperformed the other feature classes in almost all prediction pipelines including all personality traits, seeFigure 8A. In addition to the mixed-sex subject group illustrated inFigure 8A, we verified and confirmed this conclusion for the female-only and male-only subject groups. Furthermore, we also found that the majority (over 50%) of the results with prediction accuracies*r*> 0.2 collected over all cases and conditions of the pipelines were obtained from the*RCP*feature class. Interestingly, a different picture was observed for the prediction of cognition, where almost 60% of all best results were obtained from the PCA feature class. This is visualized in the Sankey plots in theSupplementary Figure S6which shows the distribution of promising results (*r*> 0.2) over the different pipeline conditions.

**Fig. 8.**
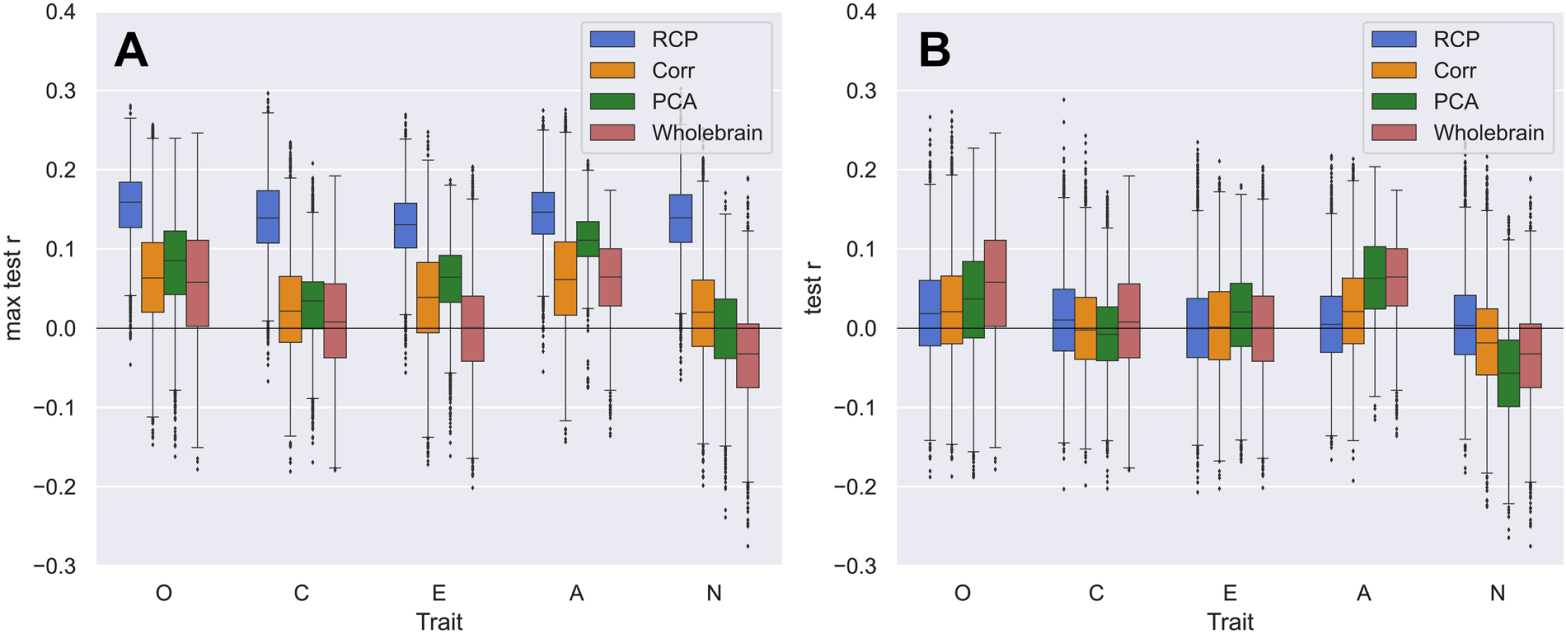
Comparing the performance of different feature classes for personality prediction from DWI data. (A) Comparing the best prediction results for different feature classes as indicated in the legend and the five traits indicated on the horizontal axis (O—Openness, C—Conscientiousness, E—Extraversion, A—Agreeableness, N—Neuroticism). Boxplots depict the distributions of the prediction accuracies calculated for 100 random data splits for CV loops and collected over all considered parcellations, all SC weights and the mixed-sex subject group. For every pipeline condition (fixed trait, weighting, parcellation, and subject group), the best result was selected across all feature selection instances of the respective feature class (RPC, number of PCs and most correlated SC edges). (B) Data presentation as in plot A. Here, the optimal feature selection instances for the different feature classes were, however, determined as a hyperparameter in the inner loop of the nested CV (see text for details).

Overall, for all different pipeline conditions, there were only a few RCPs that lead to the promising results (Fig. 2D), meaning that only a few connectivity profiles in the SC have a stronger relationship with the trait of interest, while the other connectivity profiles in the SC have little association with the personality scores. Furthermore, the predictive RCPs differ between different SC weightings and between the different personality traits as shown inFigure 5.

The above comparison, obtained by selecting the best prediction accuracy out of many others after the CV loops, that is based on the test set correlation, may serve as an idealistic upper boundary of prediction performance. As a more sophisticated approach, we ran an additional prediction analysis, where the issue of the optimal feature selection was resolved within the inner CV loop, see Methods. In more detail, we included the selection of the optimal RCP, number of PCs, and number of most correlated features as an additional hyperparameter to be optimized together with the regularization hyperparameter of the ridge regression in the inner loop of the 5-fold nested CV. The obtained results are illustrated inFigure 8Bfor the mixed-sex subject group, where we observe a drop of the prediction accuracies for almost all illustrated cases, especially for the RCP feature class. With this comparison approach, there is no clear difference between the prediction performances of different feature classes (Fig. 8B).

## 4 Discussion

### 4.1 Personality trait prediction

In the current study, we predicted the big five personality trait scores from the SC derived from dwMRI data of unrelated subjects from the HCP young adult dataset. We systematically evaluated the effect of different pipeline settings on the prediction performance including 19 cortical brain parcellations, 3 SC weightings, 3 subject groups, and 4 feature classes. For the largest of the three subject groups (mixed sex), we ran additional analysis for the prediction of a cognition target.

Overall, we find only a few cases of promising prediction performance (correlation*r*> 0.2) which are similar to values reported in the literature for personality prediction from the resting-state FC (Cai et al., 2020;Dubois et al., 2018;Hsu et al., 2018;Nostro et al., 2018). Most results, however, are centered around a correlation of zero with the mean at*r*≈ -0.003 (Fig. 3A), indicating no generalizable predictive relationship between personality traits and SC for the pipelines and data evaluated here. Considering these already very low correlations, we did not further investigate the effect of potential confounds apart from sex. The effect of sex was investigated independently by considering both a mixed-sex group and subject groups separated by sex. Nevertheless, it cannot be ruled out that other confounds such as in scanner head movement (Baum et al., 2018) or age (despite the small range from 22 to 35 years) inflated the results. This would, however, further strengthen the already observed null-findings.

While earlier studies relating dwMRI features to personality focused on TBSS and correlation analysis, the inconsistent results obtained in different analyses well support our findings. Nevertheless, for a more direct comparison, we also conducted a TBSS analysis for the data considered here. The exact method and results are given in the Supplementary Materials inTable S2. Overall, our findings for TBSS are in line with our prediction results: For most traits, there are no significant TBSS-based correlation results, and the few significant findings have low correlations with |*r*| < 0.21. Prior research based on TBSS that found significant correlations between voxel clusters and personality, in many cases reported correlations*r*≤ 0.3. Only one approach byXu and Potenza (2012)found very strong relationships between WM microstructural properties and personality with*r*’s up to 0.75 using the TBSS analysis on a small sample size of N = 56 (Xu & Potenza, 2012). A study byOoi et al. (2022)predicted different aspects of behavior as determined by a factor analysis from many behavioral scores from the SC with different weighting. The factors of*emotion*and*dissatisfaction*in the HCP dataset, as well as the*personality*factor in the ABCD dataset (Garavan et al., 2018), were predicted with correlations*r*< 0.1 for all weightings; this again is in line with the low correlations we obtained for the prediction of personality traits. Although predictions from the FC showed better results than our findings for the SC, the correlation in these cases was still limited to values below*r*= 0.29 (Cai et al., 2020;Dubois et al., 2018;Hsu et al., 2018). The only exception is the study byNostro et al. (2018)that found correlations up to*r*= 0.42 for predicting traits from certain functional brain networks. The approach byDubois et al. (2018)provided prediction accuracies for different setups and also reported negative/unsuccessful prediction accuracies for some setups for all traits except openness. This work, therefore, found the trait openness to be predicted best and most reliably from the FC, just as we did here for the SC (Fig. 3B). Considering results for brain structural measures, a review and meta-analysis byY.-W. Chen and Canli (2022)systematically evaluated research relating personality traits to brain structure, and their meta-analysis showed no replicable results for gray matter volume (GMV), cortical thickness, and surface area.

All in all, despite some promising results for certain modalities, the prediction of the big five personality traits from brain imaging data remains to be a challenging task. This is underlined by the results of our study, showing that no generalizable and clear predictive relationship between the SC and the big five personality trait scores can be found so far.

#### 4.1.1 Comparison with the prediction of cognition

To further investigate why no strong relationship between personality and the structural connections of the brain could be found, we additionally predicted a cognition score from the SC for the mixed-sex group as a comparison. Here, we found improved performance in terms of both the best performing pipelines and the average prediction accuracy compared with the prediction of personality (Fig. 4). This is in line with our expectations based on the literature that has predicted cognition targets from the SC. While there were also differences in the range of prediction correlations, most of the approaches were able to predict the cognition target to a certain extent in contrast to previous work on personality where it varied highly between approaches if a certain personality trait showed significant correlations with neuroimaging features (seeSection 1).

The improved, but still limited prediction of the cognition target indicates that the results for the personality prediction might be influenced by both inherent limitations of the standard SC (Schilling et al., 2019;Yeh et al., 2021) and reliability issues of the self-reported evaluation of personality scores themselves, which has been shown to limit brain–behavior predictions (Gell et al., 2023). Additionally, the big five personality traits represent a model of personality, and it remains unclear how close they come to actual true factors of personality and how these could ideally be measured. We, therefore, expect that further advances in both dwMRI/SC extraction and reliable estimations of the personality trait scores are necessary to find a potential connection between our personality and the structural connections of our brain. In addition, large sample sizes, enabling the use of more complex, non-linear models such as graph neural networks (Bessadok et al., 2023) might offer new opportunities to relate structural brain connectivity to personality.

Related literature on prediction of cognition from SC or FC shows diverse results, some of them are in line with what we find here. Research byOoi et al. (2022)andYeung et al. (2023)found similar correlation values for the prediction of cognition from the SC. Work byFeng et al. (2022)also showed a large range of possible prediction accuracies for different pipelines, the maximally reached values are, however, higher than in our findings (*r*up to 0.50). Reported accuracies for the prediction from the FC are mostly higher than those from the SC (Dhamala et al., 2021;Jiang et al., 2020;Ooi et al., 2022), the range of reported correlations, however, still strongly varies in*r*= [0, 0.77]. In contrast, an approach byLitwińczuk et al. (2022)finds targets for which the SC performs better than the FC for prediction (executive function, language). These results show the improved prediction of cognition targets compared with personality and the general applicability of the SC for cognition prediction across literature. The diverse results again highlight how different data, pipelines, and targets, as well as the presentation of results, can influence the prediction and complicate comparability of results.

#### 4.1.2 Prediction brain maps

Using the results from the RCP feature class pipelines, we generated cortical maps of average “local” prediction accuracy across all 19 parcellations. These maps showed distinct pictures of which RCPs contributed positively to the personality trait prediction for the five different traits. They also indicated a unique role of the different SC weightings to the prediction. Considering the overall very low prediction accuracies, we may not thoroughly interpret the RCPs leading to promising results for the different personality traits. We however highlight the potential of such maps to identify regions whose connections to other parts of the brain may have a strong link to the trait/target of interest. We expect that the connections of local voxel clusters that belong to RCPs leading to superior prediction results for many different parcellations are more strongly linked to the target. Such maps might therefore help to determine connections or regions of interest for further studies focusing on a connection between the brain SC and behavior.

### 4.2 Influence of different pipeline conditions

Despite the observed overall low prediction accuracy, we still identified differences in prediction results between distinct pipeline settings which is in line with our expectations based on the literature showing an influence of varied pipeline conditions (see e.g.,Feng et al., 2022;Yeung et al., 2023).

#### 4.2.1 Brain parcellations

There was no clear best brain parcellation for personality prediction. While there was a slight tendency toward prediction improvement with higher granularity within the same parcellation scheme, differences were not large enough to give a clear recommendation. It is also important to add that while we investigated a large number of brain parcellations, the highest granularity we used was 210 ROIs and there are significantly finer brain parcellations available (e.g., Schaefer parcellation with up 1,000 ROIs (Schaefer et al., 2018)). Considering the inherent limitations of diffusion tractography (Schilling et al., 2019;Yeh et al., 2021) and the lower resolution of diffusion MRI than other MRI modalities, the tendency toward an improvement with higher granularity might saturate or reverse at some point when continuously increasing granularity possibly leading to an optimal granularity, at least for given recording and data preprocessing settings.

Investigations byFeng et al. (2022)for prediction of cognition from the SC showed improved performance of the Human Brainnetome Atlas with 246 ROIs (Fan et al., 2016) compared with the Automated Anatomic Labeling with 90 ROIs (Tzourio-Mazoyer et al., 2002) across 2 datasets and several cognition traits. Both of these parcellations were also evaluated in this work and the improved performance of the Brainnetome atlas was confirmed for the prediction of cognition for all three SC weightings. This, however, did not hold for most configurations when predicting personality traits, showing an influence of the target. This is in line with research conducted byZhang et al. (2019)for predicting different behavioral measures from the SC. They found improvement of prediction performance for a higher granularity parcellation only for some of the traits.Dhamala et al. (2021)predict cognition from both SC and FC for a parcellation with 86 ROIs (combination of the Desikan–Killiany atlas (Desikan et al., 2006) and additional subcortical structures (Fischl et al., 2002)) and an in-house parcellation with 439 ROIs. While the prediction performance improves with a higher granularity for the FC, this does not hold for the SC for which performance does not improve or even deteriorates. This might affirm our assumption that at a certain point, higher granularity does not further improve prediction accuracy for the SC.

In an additional analysis, we also included subcortical ROIs as they are known to be linked to personality-related measures such as memory and emotion (see e.g.,Fossati, 2012;LaBar & Cabeza, 2006), and the striatum and hippocampus are some of the only ROIs consistently linked to neuroticism in fMRI studies (Lin et al., 2023). However, adding subcortical ROIs in the analysis did not improve the prediction accuracy (Supplementary Fig. S4). While these areas have been successfully related to measures of personality or related concepts, limits in tractography might have reduced this effect in the structural connectivity. The so-called bottleneck problem, where many WM bundles inhabit the same area of WM (Maier-Hein et al., 2017), might have affected subcortical areas especially strongly. Furthermore, the SNR can be weaker in subcortical regions than in cortical regions because of their larger distance to the head coil (Uğurbil et al., 2013). On average, for the parcellations considered here, subcortical regions were smaller than cortical regions, meaning that deviations in tractography can have a stronger influence on the output SC.

#### 4.2.2 SC weightings

For predicting personality, there was no global best SC weighting. However, when only considering the pipelines leading to prediction accuracies*r*> 0.2, the NOS weighting was used in a vast majority of cases (76%,Fig. 6A). For the cognition target, one could see a clear ranking of SC weightings (NOS > FA > MD) (Supplementary Fig. S5). This is consistent with the study ofYeung et al. (2023). They also found that generally the best results for prediction of sex, age, general cognition, and mental health from the SC were achieved for the NOS weighting. For predicting the general cognition factor with the BrainNetCNN, they also found a similar ranking of the SC weightings: r_MD_= 0.138, r_FA_= 0.168, and r_NOS_= 0.201. Also studies byLiu et al. (2023)andZhang et al. (2019)predicting continuous behavioral phenotypes from SCs found improved or equal performance of the NOS weighting compared with the microstructural weightings MD and FA. A study byOoi et al. (2022)on the other hand found no consistent best weighting when predicting several behavioral targets in two large-scale datasets, which demonstrates the difficulty of comparing different weightings when other pipeline conditions are set differently between studies.

Considering that for different weightings, different RCPs are predictive of a certain personality trait (Fig. 5B), it might be valuable to combine different weightings for the prediction. The higher similarity between the two maps of MD and FA compared with the more distinct pattern for the NOS map (Fig. 6B) is most likely due to the fact that MD and FA are both calculated from the eigenvalues of the diffusion tensor and are related by definition (Bihan et al., 2001), while the NOS weighting represents a completely different form of weighting. Taking this into consideration, it might be worthwhile to only consider one DTI-based microstructural weighting and combine it with the NOS weighting to enhance information capacity and improve prediction performance.

#### 4.2.3 Subject groups

A review and meta-analysis byY.-W. Chen and Canli (2022)investigating research on brain structure–personality associations found that for each trait, there was at least one study that found sex differences in the association between personality and regional gray matter volume (GMV). However, for all the traits, there was more research that examined the role of sex on the personality–brain structure relationship and found no influence of sex showing that there are overall mixed findings with the majority not showing any sex-depended associations.

Here, we did find differences in performance between the different groups (mixed sex, only male, and only female) (Fig. 7). However, there was no clear pattern and, considering the overall low correlations and relatively small sample size, it is hard to claim whether there was an influence of sex on the relationship between brain structure and personality or whether differences only arrived due to a different group of subjects independent of their sex. This would require additional investigations.

Considering the FC, not many studies investigated the role of sex on the prediction or relationship with personality traits. A study byNostro et al. (2018)found that additional functional networks predicted extraversion, neuroticism, and openness when separating subjects by sex in comparison with a mixed-sex subject group. Specifically, they for example found that openness was significantly better predicted in women than in men for the reward network. This is similar to one of our findings that openness is the only trait for which we see an improvement for the female group when separating subjects by sex (Fig. 7). This, however, only holds for the NOS weighting.

#### 4.2.4 Feature classes

Among all considered feature classes, the RCP class led to the most promising prediction performances for the personality traits (Fig. 8A). It is, however, not clear how well these RCP results generalize to other datasets. In particular, the beneficial impact of the RCP feature class on the prediction performance disappeared, when the feature selection process was optimized under the nested CV loop (Fig. 8B). This might however be connected with a relatively small sample size considered in this study, and further investigations of this issue are necessary. In general, this finding highlights the importance of out-of-sample prediction and proper assessment of the generalizability of predictive pipelines. When evaluating many different pipelines, it needs to be ensured that the best performing pipelines generalize well to new data. In addition, giving an overview over the results from all evaluated pipelines gives the reader a chance to assess how difficult it is to predict the target in general.

As mentioned above for the connectome weightings, only a few RCPs led to relatively high positive correlations between predicted and empirical personality trait scores (Fig. 2D). This may indicate that the relationship between brain structural connections and personality, to the extent that could be found here, is localized in certain connections. Single RCPs would lead to better prediction results than using the entire SC for almost all pipelines (Fig. 8A). In contrast to the personality traits, the PCA feature class led to the best results for the prediction of cognition. Here, compared with the personality traits, it also worked better to use the whole brain feature class (Supplementary Fig. S6B). Interestingly, for both the cognition and personality targets, the corr feature class did not show a particularly strong performance (Supplementary Fig. S6). In comparable literature for prediction of behavior from the FC and SC, this type of feature selection is used quite commonly through the application of the connectome-based predictive modeling (Shen et al., 2017). Despite promising prediction performance of these approaches (see e.g.,Cai et al., 2020;Jiang et al., 2018;Yoo et al., 2022), considering the results here, it might be worthwhile to investigate other feature classes as well.

### 4.3 Importance of result presentation

Publication bias led to positive and significant findings being reported and published more frequently than null-findings or non-significant results in the past (Dickersin, 2005;Easterbrook et al., 1991). An overly optimistic picture might therefore be established in the literature for certain prediction investigations when several analyses were performed and only significant or the best results were reported to enhance chances of the work being published. Presenting all the obtained prediction accuracies gives the reader a chance to assess the prediction performance that can be expected when randomly selecting a pipeline from the set of evaluated conditions. Considering the entire spectrum of the obtained results can further give a good impression on how easy or difficult it is to predict a certain target from the features of interest in general. The best results achieved by some of the pipelines evaluated here are in line with other results found in the literature. However, not presenting the vast amount of unsuccessful predictions, illustrated inFigure 3A, would leave the reader with an unrealistically good impression for the prediction of personality from the SC. Our results further show that despite using a CV prediction method to assess an out-of-sample performance, overfitting, in this case to the test set, can still occur.Figure 8clearly shows that selecting the best RCP on an independent set in the inner loop of the nested CV leads to significantly worse results than selecting it post hoc based on the calculated test set accuracies. Here, this presentation of overfitting confirms and strengthens the assumptions of null-findings for the SC–personality relationship. This also demonstrates the importance of verifying selected “good” pipeline conditions for prediction either by having them determined in the inner loop of the CV or by applying the successful pipelines to completely unseen data.

Independent of the exact targets and features used for prediction, our results show how strongly cherry-picking results obtained from several analyses might influence the perceived prediction performance and further highlight the importance of reporting all performed analyses and testing for potential overfitting.

### 4.4 Limitations

In our study, we sampled a large parameter space of different settings along the prediction pipeline that might influence the prediction results. Despite these efforts, some options that might affect the prediction performance were not evaluated in our work, a few of which we would like to point out here.

In this study, we used a ridge regression model for prediction. Previous research shows minimal differences in prediction accuracy between linear models and non-linear deep learning models for behavioral phenotypes from the SC and FC (Schulz et al., 2020;Yeung et al., 2023). Furthermore,Cui and Gong (2018)compared six regression algorithms for predicting behavioral variables from resting-state FC features and found that four algorithms, including ridge regression, performed similarly well, with ridge regression having the lowest computational burden.Feng et al. (2022)reported improved prediction accuracy using deep learning for age and cognition from the SC, though classical models still performed comparably strong. Based on these findings and its low computational cost, we chose ridge regression, which enabled the comparison of other pipeline parameters. The literature suggests that the low prediction accuracy for personality is unlikely due to the choice of algorithm, although we did not directly compare it with deep learning or non-linear models.

The structural connectome (SC) often contains false positive streamlines or connections, which previous approaches have addressed by thresholding SCs to retain only connections present in a large fraction of subjects (Feng et al., 2022;Yeung et al., 2023). Here, we did not apply such a thresholding to the SCs as it might have a similar effect as the applied corr feature class and there is no consensus on the best method or threshold. A past study showing an influence (both positive and negative) of thresholding on prediction results from the SC (Litwińczuk et al., 2022) underlines the importance of such a consideration. On the other hand, the heterogeneous findings of the above work and diverse thresholding possibilities warrant an in-depth examination which is outside the scope of this project and would require more stable results than we are seeing here.

We chose not to normalize the SC weighted by NOS based on ROI size, an approach used to reduce the bias toward larger ROIs being connected by more streamlines (Jones et al., 2013). We expect this to not strongly affect research on inter-individual differences as the same parcellation was used across subjects and, therefore, all instances of a given feature would be based on the same scaling across subjects. However, there is no conclusive evidence on whether normalization affects prediction outcomes, as some studies have implemented it (Dhamala et al., 2021;Feng et al., 2022), while others have not (Yeung et al., 2023;Yoo et al., 2022). Therefore, the potential impact of normalization cannot be entirely dismissed and warrants further investigation.

In our work, we show that personality traits cannot be reliably predicted from the SC. Despite the results for personality prediction from the SC, which show poorer performance than personality prediction from the FC, the SC has been successfully used for predicting phenotypes in other contexts (see e.g.,Feng et al., 2022;Yeung et al., 2023) which resonates well with our improved prediction performance of cognition and shows that there is valuable behavioral information contained in the structural brain connections. Other approaches have further shown that combining information from dwMRI and fMRI can boost the prediction accuracies beyond what separate modalities can do (Dhamala et al., 2021;Litwińczuk et al., 2022;Rasero et al., 2021). This demonstrates that structural connectivity data hold value in addition to functional data for the analysis of brain–behavior relationships. While combining features from multiple modalities can boost the prediction performance, how to best combine them remains an open question. Furthermore, multimodal setups increase the number of features which can lead to overfitting and, therefore, require a higher sample size for the analysis (J. Chen et al., 2023).

In summary, more research is necessary to determine how to improve the reliability of both prediction target traits and input brain features to maximize what we can learn about how behavior is linked to the brain. Our results highlight the crucial impact of the overall pipeline on prediction accuracies and the need to be careful when linking personality to the SC.

## 5 Summary

In the present study, we systematically evaluated different pipeline conditions for the prediction of the big five personality trait scores from the SC. Despite the large variability in pipelines, we found no linear generalizable relationship between personality trait scores and the structural connectome derived from dwMRI data. Only a few pipelines could be found that lead to promising results while still of low correlations between empirical and predicted scores. Considering the vast majority of unsuccessful predictions and the comparably small sample sizes, we do not expect the more successful setups of the prediction pipelines to generalize well to new unseen data. Comparisons with the results for prediction of a cognition target indicate that there might be limitations considering the reliability of the personality target as well as the reliability of current tractography and SC reconstruction algorithms. We expect that larger advances in the reliability of both trait scores and dwMRI/SC calculation as well as larger samples will be necessary to uncover a potential connection between the structural connections of the brain and personality traits. Overall, it became apparent that all different design choices we evaluated in the process of calculating the SC and predicting a behavioral target from it influenced the prediction outcome. Apart from comparing obtained prediction accuracies for different combinations of pipeline conditions, we calculated and compared prediction brain maps of local prediction accuracy. We found distinct prediction brain maps for different personality trait scores and highlighted the potential of such maps to determine regions whose connections to the rest of the brain resemble good features for predicting a certain target trait. Distinct maps could also be found for different connectome weightings. While the maps of the two microstructural weightings were quite similar, the map of the NOS weighting showed a distinct pattern. Even though the NOS weighting outperformed microstructural properties as SC weighting for both the cognition and personality variables, it might be beneficial to combine the NOS weighting with a microstructural weighting for enhanced information capacity and possibly better prediction.

## Supplementary Material

Supplementary Material

## Data Availability

The anonymized data from the Human Connectome Project used for this analysis are publicly available from ConnectomeDB. The structural connectivity pipeline (https://github.com/ameliecr/SC-calculation-pipeline) and the code used for prediction (https://github.com/ameliecr/SCandBigFive) are available on GitHub.
