# Supplementary material for: Weak and unstable prediction of personality from the structural connectome
Source: Imaging Neurosci (Camb). 2025 Jan 3;3:imag_a_00416. doi: 10.1162/imag_a_00416 (PMC12319827; doi:10.1162/imag_a_00416)
Supplement: Supplementary Material [file imag_a_00416-supp.pdf]

Supplementary Materials

for the manuscript

Weak and Unstable Prediction of Personality from the Structural Connectome

by

Amelie Rauland[1,2], Kyesam Jung[2,3], Theodore D. Satterthwaite[4,5,6], Matthew Cieslak[4,5], Kathrin Reetz[7,8], Simon B. Eickhoff[2,3], Oleksandr V. Popovych[2,3]

[1] Department of Psychiatry, Psychotherapy and Psychosomatics, RWTH Aachen, Aachen, Germany
[2] Institute of Neuroscience and Medicine, Brain and Behaviour (INM-7), Research Centre Jülich, Jülich, Germany
[3] Institute for Systems Neuroscience, Medical Faculty, Heinrich-Heine University Düsseldorf, Düsseldorf, Germany
[4] Lifespan Informatics and Neuroimaging Center, University of Pennsylvania Perelman School of Medicine, Philadelphia Pennsylvania, United States
[5] Department of Psychiatry, University of Pennsylvania Perelman School of Medicine, Philadelphia Pennsylvania, United States
[6] Penn-CHOP Lifespan Brain Institute, Philadelphia Pennsylvania, United States
[7] Department of Neurology, RWTH Aachen University, Aachen, Germany
[8] JARA-BRAIN Institute of Molecular Neuroscience and Neuroimaging, Forschungszentrum Jülich GmbH and RWTH Aachen University,
Aachen, Germany

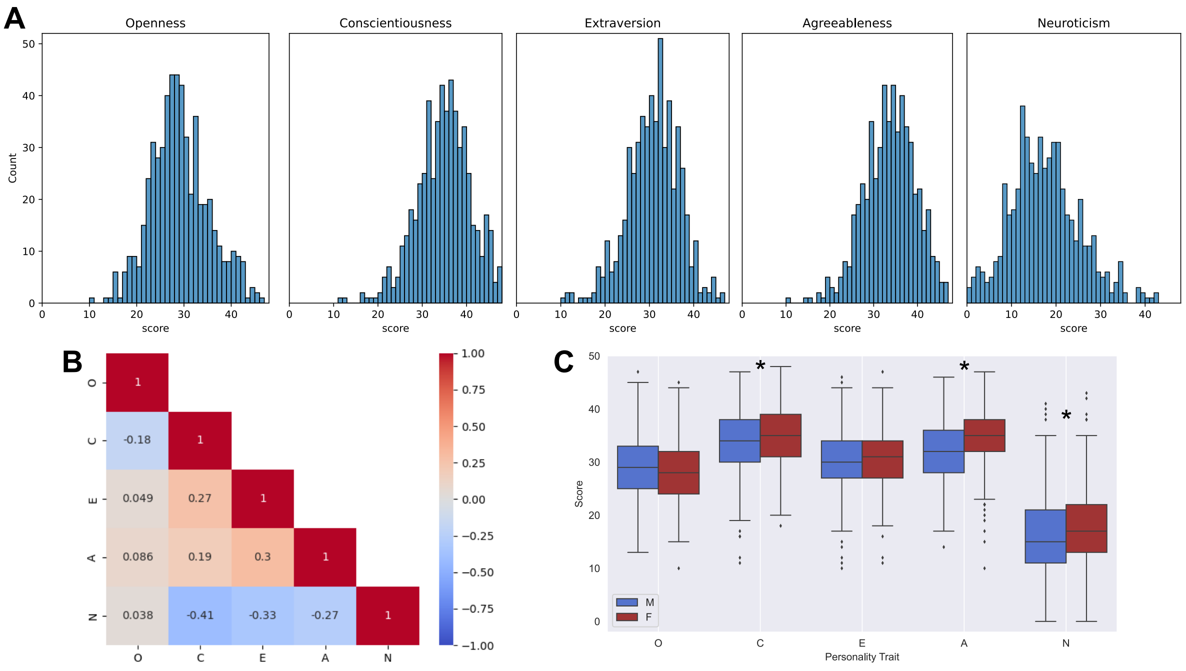

Figure S1 **Supplementary information on the personality trait scores.** Only scores of subjects used in the analysis were used for the preparation of the figures. **A** The distribution of the big five personality trait scores as obtained through the NEO-FFI questionnaire. **B** The correlation between the different trait scores (O - Openness, C - Conscientiousness, E - Extraversion, A - Agreeableness, N - Neuroticism) **C** Comparison of trait distributions between males and females. For traits marked with an asterisk, the difference between the distributions of male and female trait scores was statistically significant with $p < 0.05$.

Name	# parcels	Based on	Parcellated by	Refs.
MIST	31	functional MRI	Clustering based on connectivity properties	Urchs et al., 2019
	56			
	103			
	167			
Craddock	38	functional MRI	Clustering based on connectivity properties	Craddock et al., 2012
	56			
	108			
	160			
Shen 2013	79	functional MRI	Clustering based on connectivity properties	Shen et al., 2013
	156			
Schaefer	100	functional MRI	Clustering + boudary detection based on connectivity properties	Schaefer et al., 2018
	200			
Harvard-Oxford	48	structural MRI	Boundary detection based on local properties	Desikan et al., 2006; Frazier et al., 2005; Goldstein et al., 2007; Makris et al., 2006
	96			
Desikan-Killiany	70	structural MRI	Boundary detection based on local properties	(Desikan et al., 2006)
Economo-Koskinas	86	histology (structure)	Boundary detection based on local properties	(Economo & Koskinas, n.d.); Scholtens et al., 2018;
AAL (version 2)	92	structural MRI	Boundary detection based on local properties	Rolls et al., 2015; Tzourio-Mazoyer et al., 2002
Destrieux	150	structural MRI	Boundary detection based on local properties	Destrieux et al., 2010
Brainnetome	210	structural MRI	Clustering based on connectivity properties	Fan et al., 2016

Table S1 **Overview of the used brain parcellation schemes** with the number of parcels after image processing, details on their derivation and associated publications. This table is adapted from (Domhof et al., 2021).

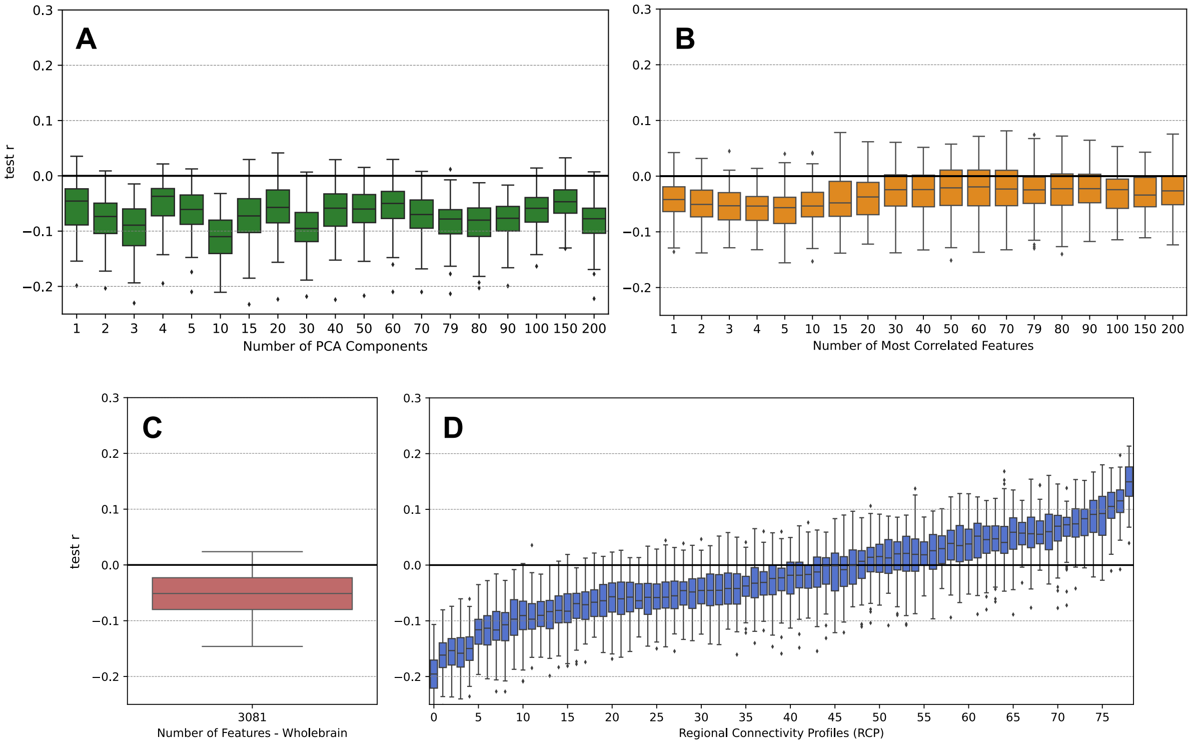

Figure S2 **Examples of prediction results for all four feature classes.** The calculations were performed by the pipeline with the Shen atlas (79 ROIs), NOS weighting, mixed-sex subject group, and the trait neuroticism. The box plots show the distributions of the prediction accuracy as given by Pearson's correlation between the predicted and empirical personality scores obtained for the test sets over 100 random splits of the data of the 5-fold cross-validation. The prediction results are depicted for **A** the PCA feature class with different numbers of PCA components, **B** the corr feature class with different numbers of the most correlated features, **C** the whole-brain feature class and **D** the Regional Connectivity Profile (RCP*)* feature class for different brain regions, i.e., rows of the SC matrix. RCPs are sorted by the mean prediction accuracy. The respective conditions of the feature selection are indicated on the horizontal axes with the non-linear scaling in plots A and B.

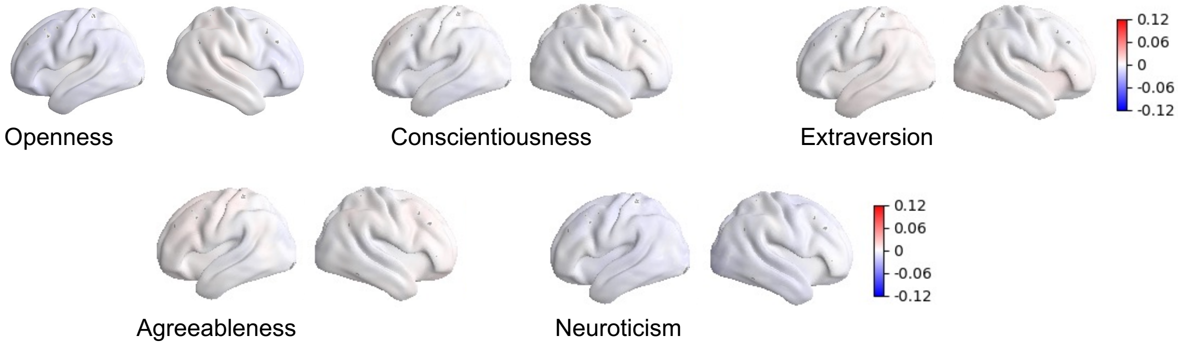

Figure S3 **Prediction brain maps for permutation test predictions.** The prediction accuracy (correlation) of the RCP feature class was assigned to all voxels of the respective brain regions and averaged over all considered parcellations for fixed other conditions of the prediction pipeline (see Methods). The brain maps are illustrated for the five different personality traits indicated in the plots as prediction targets obtained for the mixed sex subject group and the NOS SC weighting for randomly permuted target values. The visualization of the maps was created using the neuromaps toolbox (Markello et al., 2022) including the volume-to-surface transformations as proposed and defined in (Buckner et al., 2011), (Wu et al., 2018).

Analysis including subcortical ROIs

In our main analysis, all parcellations only included cortical ROIs. As subcortical ROIs have been linked to personality related measures such as memory and emotion (see e.g. LaBar & Cabeza, 2006), we performed a supplementary analysis including subcortical ROIs in the considered brain parcellations. The analysis was repeated for all 19 parcellations, three SC weightings, four feature classes, three subject groups and five personality traits. For the atlases that originally included subcortical regions, we used a subcortical GM mask from freesurfer (Fischl, 2012) to extract these regions and add them to the cortical parcellation. For the atlases that did not include subcortical regions, we added the subcortical labels from the Melbourne atlas (Tian et al., 2020). Where there was an overlap between the cortical and subcortical ROIs we removed the corresponding part from the subcortical ROI to keep the cortical parcellation unchanged. While in the original analysis all parcellations included cortical ROIs only but differed in how the cortical ROIs were defined, in the additional analysis all parcellations contained cortical and subcortical ROIs and differed in how both cortical and subcortical ROIs were defined.

The prediction results of the new analysis including subcortical regions are summarized in Fig. S4A, where the distribution of mean test correlations in orange from the new analysis can be compared to the distribution in blue of the mean test correlations from the original analysis. There is no improvement or deterioration of the prediction accuracy by adding the subcortical ROIs to the parcellations. Both the blue and orange distributions are centered around zero with the mean value being slightly below zero ($mean_{cort}$ = -0.003, $mean_{cort+subcort}$ = -0.004). The effect size of the difference between the distributions as determined by Cohen's d is very small (d = 0.0158) meaning the distributions are very similar. This finding also holds when comparing the distributions of the top and bottom 5% of both result distributions to assess the effect size of the difference between both distributions for the best and the worst results. For the top 5% of the results Cohen's d between the two distributions is d = 0.0515 for the bottom 5% of the results d = 0.0745 showing that the distributions are not only similar on average but also when only considering the best and worst results. While the very best result improved from $r_{max,cort}$ = 0.307 to $r_{max,cort+subcort}$ = 0.329 when including subcortical ROIs the number of results with r > 0.2 decreased from $n_{r>0.2,cort}$ = 262 to $n_{r>0.2,cort+subcort}$ = 247. Figure S4B shows the results of the new analysis (including subcortical ROIs) separated by traits. As for the original analysis, we see a slightly better prediction performance for the Openness trait compared to the other traits (comp. Fig. 3B). There is an improvement of the best result for the traits openness ($r_{max,cort+subcort}$=0.329 vs. $r_{max,cort}$=0.307) and agreeableness ($r_{max,cort+subcort}$=0.254 vs. $r_{max,cor}$t=0.226) when including subcortical ROIs while no strong change can be observed for the traits extraversion, conscientiousness and neuroticism. Figure S4C shows the results from the RCP feature class separated depending on if the ROI belonging to the RCP is cortical or subcortical. Both distributions of the prediction accuracy are still centered around zero and there is no improvement for results predicted by RCPs of subcortical ROIs compared to cortical ROIs. The effect size between the two distributions in terms of Cohen's d is very small (d = 0.054) showing that the distributions are very similar.

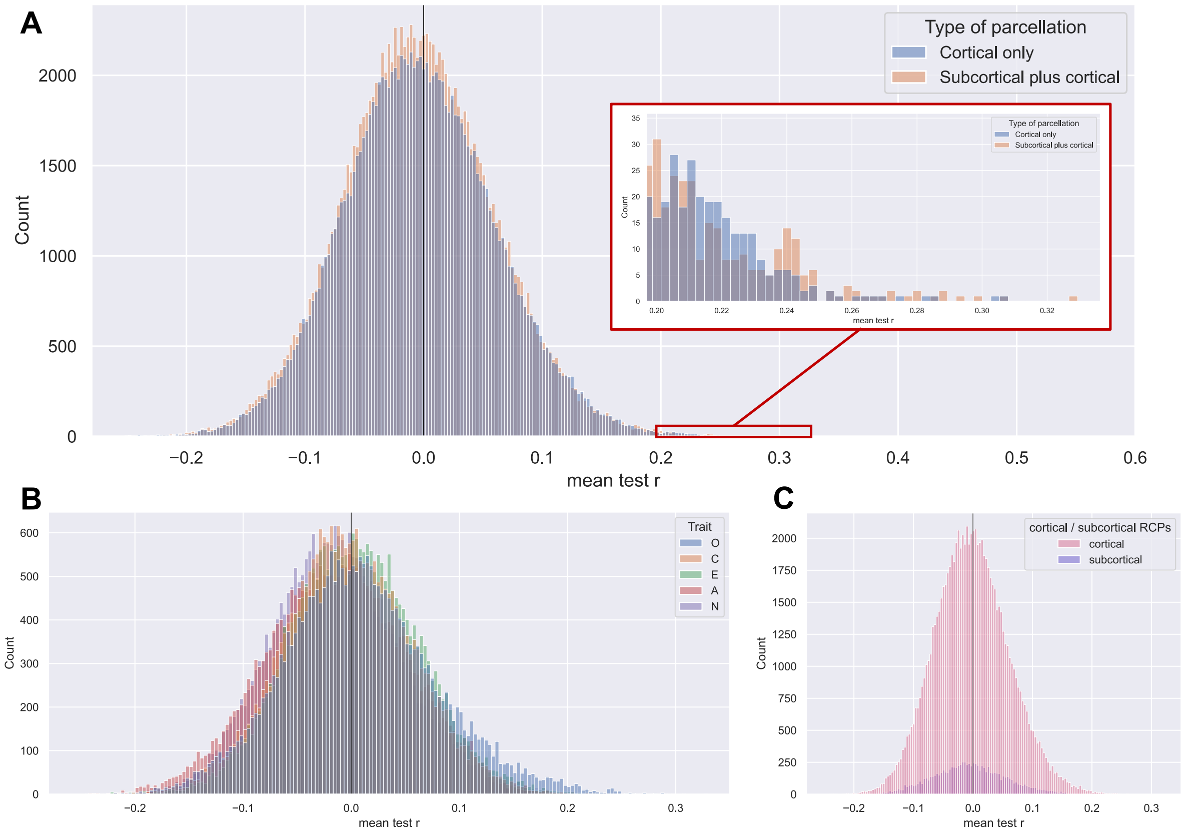

Figure S4 **Prediction results for including subcortical regions in the brain parcellations. A** Histogram showing the overall distribution of the prediction accuracy given by the mean correlation r between empirical and predicted personality traits obtained for the test sets and averaged across 100 random subject splits for cross-validation. The prediction results are collected from all different pipelines with varying parcellation, SC weighting, subject group, personality trait, and feature class as well as all conditions of the feature selection. The latter includes different numbers of PCA components (19 options), different numbers of the most correlated SC edges (19 options) and all RCPs (number of options depended on the granularity of the parcellation). The orange distribution shows the results using parcellations including subcortical ROIs, and the blue distribution shows the results using parcellations with only cortical ROIs. The selected and enlarged area of the histogram shows the cases of r > 0.2. **B** Five overlaid histograms of the prediction results of the orange distribution from plot (A) separated by the five different personality traits as indicated in the legend. The trait abbreviations are as follows O: Openness, C: Conscientiousness, E: Extraversion, A: Agreeableness, N: Neuroticism. **C** Two overlaid histograms of a subset of the prediction results of the orange distribution from plot (A). The displayed results are from the RCP feature class only. Results from RCPs belonging to subcortical regions are displayed in purple, and the results from RCPs belonging to cortical regions are displayed in purple.

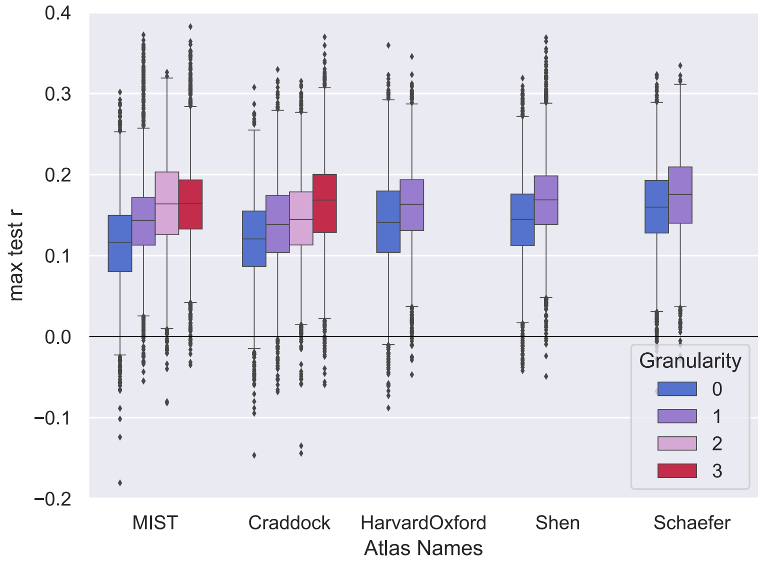

Figure S5 **Comparison of brain parcellation granularity.** The figure compares the distributions of the maximum prediction correlations from pipelines using the RCP feature class for brain parcellations with the same parcellation scheme at different granularities. The distributions contain results from all personality traits and all connectome weightings.

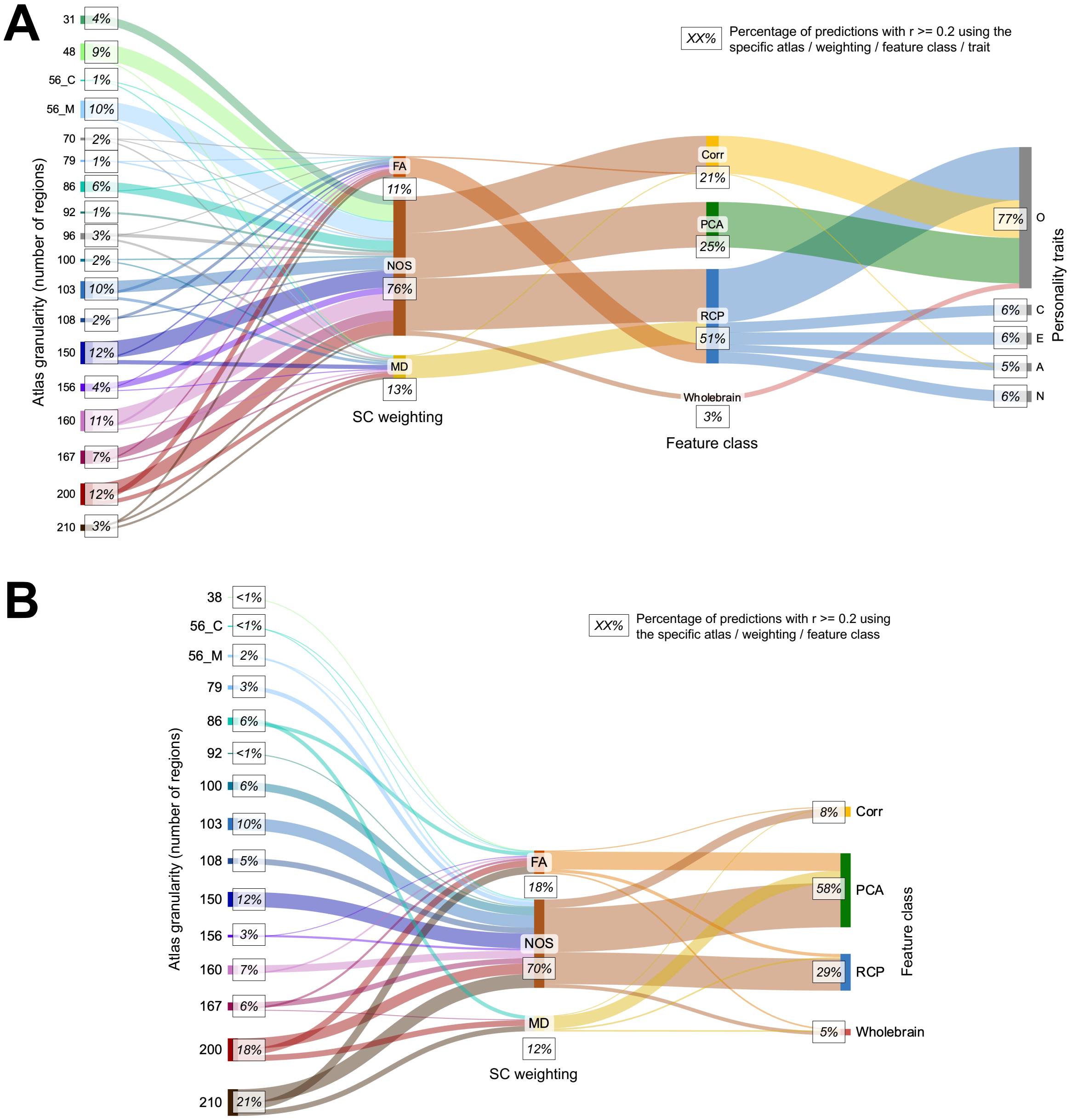

Figure S6 **Sankey graphs of the best pipelines leading to test set correlations r > 0.2.** On the left are the different parcellation schemes, followed by the three different SC weightings and then the feature selection methods and the different feature selections / representations. The larger a node, the more often it was part of a pipeline leading to a test set correlation > 0.2. The percentage in the box shows which percent of the considered prediction pipelines used this setting compared to other settings within the same category. Some atlases are not displayed in the plot since no pipelines using these parcellations led to predictions with r > 0.2. The exact combinations of settings leading to prediction correlations r > 0.2 can be found in the csv files in the project's GitHub repository (https://github.com/ameliecr/SCandBigFive). Diagram created using SankeyMATIC. **A** For personality trait scores (O: Openness, C: Conscientiousness, E: Extraversion, A: Agreeableness, N: Neuroticism). Here, the total number of predictions with r > 0.2 = 262 (out of the overall number of 123,615 of prediction results). **B** For the cognition target. Here, the total number of predictions with r > 0.2 = 239 (out of the overall number of 8241 of prediction results).

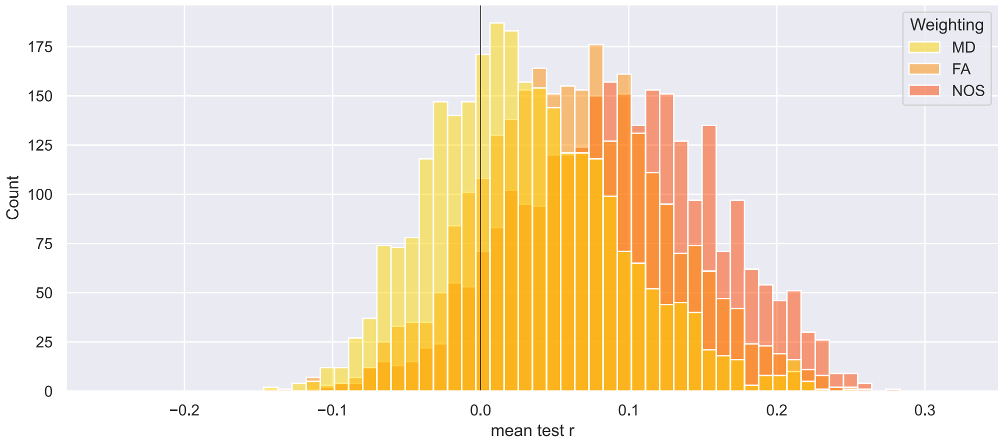

Figure S7 Distribution of mean test set correlations for the prediction of cognition separated by connectome weighting. Effect size between the distributions was calculated as Cohen's d: d_{MD-FA} = 0.55, d_{FA-NOS} = 0.46, d_{MD-NOS} = 0.99.

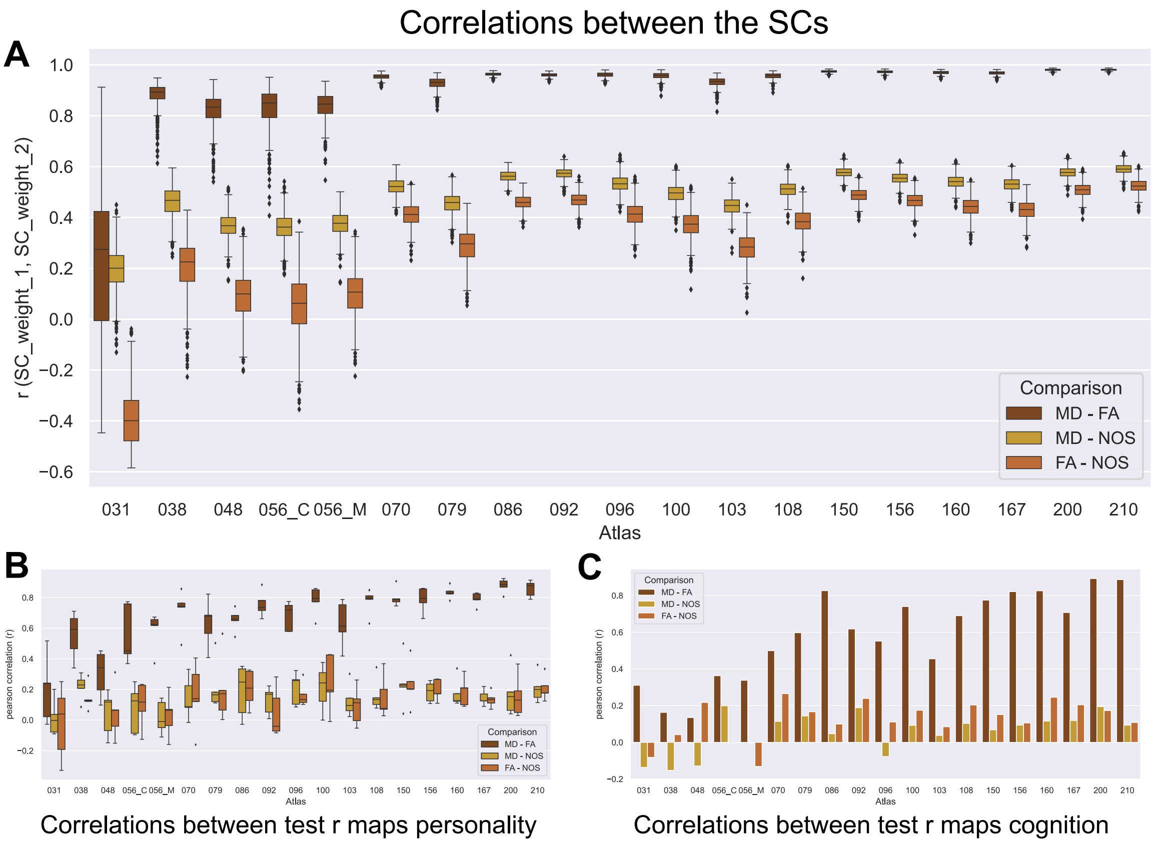

Figure S8 **A** Pearson correlation r between the SCs with different weightings for every individual cortical brain atlas. The correlation was calculated for each subject included in the study (n=560), leading to a distribution of correlation values. **B** Pearson correlation r between the test r maps for different connectome weightings obtained from the RCP feature representation predictions of personality trait scores for individual cortical brain parcellations. The distributions each contain 15 values (three different subject groups x five personality trait scores). **C** Pearson correlation r between the test r maps obtained from the RCP feature representation predictions of a cognition score for individual cortical brain parcellations. Here, there is only one correlation value and not a distribution of correlations as these predictions were only performed for the mixed sex subject group and one composite cognition score.

TBSS Analysis

In addition to our prediction analysis, we ran a TBSS analysis for our data to obtain results which can directly be compared to published findings. The analysis was conducted for all three subject groups (mixed-sex, male only and female only) for the fractional anisotropy (FA) images. We used the FA images calculated with MRtrix3 (Tournier et al., 2019) for the structural connectome (SC) construction and then used the implementation of the TBSS analysis provided by FSL (Smith et al., 2004). All FA images were aligned to a standard space by applying a non-linear registration. Subsequently the mean across all aligned FA images was calculated to extract the mean FA skeleton from it. The skeleton was thresholded at a value of 0.2 and only voxels that were part of the thresholded skeleton mask were used in the following statistical analysis. A detailed description of the algorithm up to this point can be found in Ref. (Smith et al., 2007).

Voxel-wise cross-subject statistical analysis between FA and the different personality traits were performed with the *randomize* tool in FSL (Smith et al., 2004) which implemented a general linear model in conjunction with, here, 10,000 Monte Carlo simulations. Threshold-free cluster enhancement (TFCE) (Smith & Nichols, 2009) was applied for final voxel-wise inference. All personality trait scores were demeaned before the analysis and both positive and negative associations between trait scores and FA values were investigated.

Statistically significant findings are listed in Table S2. For each subject group, only one respective trait was found to significantly correlate with a certain voxel cluster. For the mixed-sex subject group openness was significantly positively correlated with the FA values in one cluster of 45 voxels, for the female only subject group neuroticism was significantly negatively correlated with the FA in a cluster of 31 voxels, and for the male only subject group a cluster of 12 voxels was significantly correlated with conscientiousness trait scores. The table further gives information on the mean and max absolute Pearson's correlation across voxels in the cluster as well as the MNI coordinates of the center of gravity (COG) of the cluster. For all other traits in each of the subject groups, there were no significant findings. The previous literature relating personality to measures extracted from dwMRIs via TBSS only investigated mixed-sex subject groups. None of these approaches found only significant results for the openness trait as we did here for the mixed sex subject group: Several approaches found no significant results for openness ((Avinun et al., 2020), (Booth et al., 2014), (Rodriguez et al., 2019), (Sanjari Moghaddam et al., 2020)) and another approach found significant results for openness but also for agreeableness and neuroticism (Marc N. Potenza, 2012). Jung et al. (Jung et al., 2010) found significant findings only for openness. However, they did not investigate any of the other traits. Overall, our findings only comprise comparably small clusters of voxels with low maximum absolute correlations for a few different traits for different subject groups. This therefore goes well in line with our analyses demonstrating weak and unstable prediction of personality traits from SC.

					MNI152 coordinates of COG		
Subject Group	Trait	Cluster Size	Mean r	Max |r|	X	Y	Z
All	O	45	0.12	0.18	-43	-60	-5
F	N	31	-0.08	|-0.21|	44	-59	-7
M	C	12	0.14	0.14	37	17	37

Table S2 **Significant results for the TBSS analysis.** Results are separated by subject groups (all – mixed-sex, F – females only, M – males only). Given is the trait for which significant results were found (O – Openness, N – Neuroticism, C – Conscientiousness), the cluster size in number of voxels, the mean Pearson's correlation between

the FA values and demeaned personality trait scores across all voxels in the cluster, the maximum Pearson's correlation of all voxels in the cluster and the MNI coordinates of the center of gravity of the cluster.

References

Avinun, R., Israel, S., Knodt, A. R., & Hariri, A. R. (2020). Little evidence for associations between the Big Five personality traits and variability in brain gray or white matter. *NeuroImage*, *220*, 117092. https://doi.org/10.1016/j.neuroimage.2020.117092

Booth, T., Mõttus, R., Corley, J., Gow, A. J., Henderson, R. D., Maniega, S. M., Murray, C., Royle, N. A., Sprooten, E., Hernández, M. C. V., Bastin, M. E., Penke, L., Starr, J. M., Wardlaw, J. M., & Deary, I. J. (2014). Personality, health, and brain integrity: The Lothian Birth Cohort Study 1936. *Health Psychology*, *33*(12), 1477–1486. https://doi.org/10.1037/hea0000012

Buckner, R. L., Krienen, F. M., Castellanos, A., Diaz, J. C., & Yeo, B. T. T. (2011). The organization of the human cerebellum estimated by intrinsic functional connectivity. *Journal of Neurophysiology*, *106*(5), 2322–2345. https://doi.org/10.1152/jn.00339.2011

Craddock, R. C., James, G. A., Holtzheimer, P. E., Hu, X. P., & Mayberg, H. S. (2012). A whole brain fMRI atlas generated via spatially constrained spectral clustering. *Human Brain Mapping*, *33*(8), 1914–1928. https://doi.org/10.1002/hbm.21333

Desikan, R. S., Ségonne, F., Fischl, B., Quinn, B. T., Dickerson, B. C., Blacker, D., Buckner, R. L., Dale, A. M., Maguire, R. P., Hyman, B. T., Albert, M. S., & Killiany, R. J. (2006). An automated labeling system for subdividing the human cerebral cortex on MRI scans into gyral based regions of interest. *NeuroImage*, *31*(3), 968–980. https://doi.org/10.1016/j.neuroimage.2006.01.021

Destrieux, C., Fischl, B., Dale, A., & Halgren, E. (2010). Automatic parcellation of human cortical gyri and sulci using standard anatomical nomenclature. *NeuroImage*, *53*(1), 1–15. https://doi.org/10.1016/j.neuroimage.2010.06.010

Domhof, J. W. M., Jung, K., Eickhoff, S. B., & Popovych, O. V. (2021). Parcellation-induced variation of empirical and simulated brain connectomes at group and subject levels. *Network Neuroscience*, *5*(3), 798–830. https://doi.org/10.1162/netn_a_00202

Economo, C., & Koskinas, G. (n.d.). *Die Cytoarchitektonik der Hirnrinde des erwachsenen Menschen*. Retrieved August 8, 2024, from https://www.semanticscholar.org/paper/Die-Cytoarchitektonik-der-Hirnrinde-des-erwachsenen-Economo-Koskinas/6a221e8fc0d66292d9c90f7f94c3cb83866427c6

Fan, L., Li, H., Zhuo, J., Zhang, Y., Wang, J., Chen, L., Yang, Z., Chu, C., Xie, S., Laird, A. R., Fox, P. T., Eickhoff, S. B., Yu, C., & Jiang, T. (2016). The Human Brainnetome Atlas: A New Brain Atlas Based on Connectional Architecture. *Cerebral Cortex (New York, N.Y.: 1991)*, *26*(8), 3508–3526. https://doi.org/10.1093/cercor/bhw157

Fischl, B. (2012). FreeSurfer. *NeuroImage*, *62*(2), 774–781. https://doi.org/10.1016/j.neuroimage.2012.01.021

Fossati, P. (2012). Neural correlates of emotion processing: From emotional to social brain. *European Neuropsychopharmacology*, *22*, S487–S491. https://doi.org/10.1016/j.euroneuro.2012.07.008

Frazier, J. A., Chiu, S., Breeze, J. L., Makris, N., Lange, N., Kennedy, D. N., Herbert, M. R., Bent, E. K., Koneru, V. K., Dieterich, M. E., Hodge, S. M., Rauch, S. L., Grant, P. E., Cohen, B. M., Seidman, L. J., Caviness, V. S., & Biederman, J. (2005). Structural brain magnetic resonance imaging of limbic and thalamic volumes in pediatric bipolar disorder. *The American Journal of Psychiatry*, *162*(7), 1256–1265. https://doi.org/10.1176/appi.ajp.162.7.1256

Goldstein, J. M., Seidman, L. J., Makris, N., Ahern, T., O'Brien, L. M., Caviness, V. S., Kennedy, D. N., Faraone, S. V., & Tsuang, M. T. (2007). Hypothalamic abnormalities

in schizophrenia: Sex effects and genetic vulnerability. *Biological Psychiatry*, *61*(8), 935–945. https://doi.org/10.1016/j.biopsych.2006.06.027

Jung, R. E., Grazioplene, R., Caprihan, A., Chavez, R. S., & Haier, R. J. (2010). White Matter Integrity, Creativity, and Psychopathology: Disentangling Constructs with Diffusion Tensor Imaging. *PLOS ONE*, *5*(3), e9818. https://doi.org/10.1371/journal.pone.0009818

LaBar, K. S., & Cabeza, R. (2006). Cognitive neuroscience of emotional memory. *Nature Reviews Neuroscience*, *7*(1), 54–64. https://doi.org/10.1038/nrn1825

Makris, N., Goldstein, J. M., Kennedy, D., Hodge, S. M., Caviness, V. S., Faraone, S. V., Tsuang, M. T., & Seidman, L. J. (2006). Decreased volume of left and total anterior insular lobule in schizophrenia. *Schizophrenia Research*, *83*(2–3), 155–171. https://doi.org/10.1016/j.schres.2005.11.020

Marc N. Potenza, J. X. (2012). White matter integrity and five-factor personality measures in healthy adults. *NeuroImage*, *59*(1), 800–807. https://doi.org/10.1016/j.neuroimage.2011.07.040

Markello, R. D., Hansen, J. Y., Liu, Z.-Q., Bazinet, V., Shafiei, G., Suárez, L. E., Blostein, N., Seidlitz, J., Baillet, S., Satterthwaite, T. D., Chakravarty, M. M., Raznahan, A., & Misic, B. (2022). neuromaps: Structural and functional interpretation of brain maps. *Nature Methods*, *19*(11), 1472–1479. https://doi.org/10.1038/s41592-022-01625-w

Rodriguez, C., Jagadish, A. K., Meskaldji, D.-E., Haller, S., Herrmann, F., Van De Ville, D., & Giannakopoulos, P. (2019). Structural Correlates of Personality Dimensions in Healthy Aging and MCI. *Frontiers in Psychology*, *9*. https://www.frontiersin.org/articles/10.3389/fpsyg.2018.02652

Rolls, E. T., Joliot, M., & Tzourio-Mazoyer, N. (2015). Implementation of a new parcellation of the orbitofrontal cortex in the automated anatomical labeling atlas. *NeuroImage*, *122*, 1–5. https://doi.org/10.1016/j.neuroimage.2015.07.075

Sanjari Moghaddam, H., Mehrabinejad, M.-M., Mohebi, F., Hajighadery, A., Maroufi, S. F., Rahimi, R., & Aarabi, M. H. (2020). Microstructural white matter alterations and personality traits: A diffusion MRI study. *Journal of Research in Personality*, *88*, 104010. https://doi.org/10.1016/j.jrp.2020.104010

Schaefer, A., Kong, R., Gordon, E. M., Laumann, T. O., Zuo, X.-N., Holmes, A. J., Eickhoff, S. B., & Yeo, B. T. T. (2018). Local-Global Parcellation of the Human Cerebral Cortex from Intrinsic Functional Connectivity MRI. *Cerebral Cortex (New York, N.Y.: 1991)*, *28*(9), 3095–3114. https://doi.org/10.1093/cercor/bhx179

Scholtens, L. H., de Reus, M. A., de Lange, S. C., Schmidt, R., & van den Heuvel, M. P. (2018). An MRI Von Economo – Koskinas atlas. *NeuroImage*, *170*, 249–256. https://doi.org/10.1016/j.neuroimage.2016.12.069

Shen, X., Tokoglu, F., Papademetris, X., & Constable, R. T. (2013). Groupwise whole-brain parcellation from resting-state fMRI data for network node identification. *NeuroImage*, *82*, 403–415. https://doi.org/10.1016/j.neuroimage.2013.05.081

Smith, S. M., Jenkinson, M., Woolrich, M. W., Beckmann, C. F., Behrens, T. E. J., Johansen-Berg, H., Bannister, P. R., De Luca, M., Drobnjak, I., Flitney, D. E., Niazy, R. K., Saunders, J., Vickers, J., Zhang, Y., De Stefano, N., Brady, J. M., & Matthews, P. M. (2004). Advances in functional and structural MR image analysis and implementation as FSL. *NeuroImage*, *23*, S208–S219. https://doi.org/10.1016/j.neuroimage.2004.07.051

Smith, S. M., Johansen-Berg, H., Jenkinson, M., Rueckert, D., Nichols, T. E., Miller, K. L., Robson, M. D., Jones, D. K., Klein, J. C., Bartsch, A. J., & Behrens, T. E. J. (2007). Acquisition and voxelwise analysis of multi-subject diffusion data with Tract-Based Spatial Statistics. *Nature Protocols*, *2*(3), Article 3. https://doi.org/10.1038/nprot.2007.45

Smith, S. M., & Nichols, T. E. (2009). Threshold-free cluster enhancement: Addressing problems of smoothing, threshold dependence and localisation in cluster inference. *NeuroImage*, *44*(1), 83–98. https://doi.org/10.1016/j.neuroimage.2008.03.061

Tian, Y., Margulies, D. S., Breakspear, M., & Zalesky, A. (2020). Topographic organization of the human subcortex unveiled with functional connectivity gradients. *Nature Neuroscience*, *23*(11), 1421–1432. https://doi.org/10.1038/s41593-020-00711-6

Tournier, J.-D., Smith, R., Raffelt, D., Tabbara, R., Dhollander, T., Pietsch, M., Christiaens, D., Jeurissen, B., Yeh, C.-H., & Connelly, A. (2019). MRtrix3: A fast, flexible and open software framework for medical image processing and visualisation. *NeuroImage*, *202*, 116137. https://doi.org/10.1016/j.neuroimage.2019.116137

Tzourio-Mazoyer, N., Landeau, B., Papathanassiou, D., Crivello, F., Etard, O., Delcroix, N., Mazoyer, B., & Joliot, M. (2002). Automated anatomical labeling of activations in SPM using a macroscopic anatomical parcellation of the MNI MRI single-subject brain. *NeuroImage*, *15*(1), 273–289. https://doi.org/10.1006/nimg.2001.0978

Urchs, S., Armoza, J., Moreau, C., Benhajali, Y., St-Aubin, J., Orban, P., & Bellec, P. (2019). MIST: A multi-resolution parcellation of functional brain networks. *MNI Open Research*, *1*, 3. https://doi.org/10.12688/mniopenres.12767.2

Wu, J., Ngo, G. H., Greve, D., Li, J., He, T., Fischl, B., Eickhoff, S. B., & Yeo, B. T. T. (2018). Accurate nonlinear mapping between MNI volumetric and FreeSurfer surface coordinate systems. *Human Brain Mapping*, *39*(9), 3793–3808. https://doi.org/10.1002/hbm.24213
